# Effects of Cracking Test Conditions on Estimation Uncertainty for Weibull Parameters Considering Time-Dependent Censoring Interval

**DOI:** 10.3390/ma10010003

**Published:** 2016-12-23

**Authors:** Jae Phil Park, Chanseok Park, Jongweon Cho, Chi Bum Bahn

**Affiliations:** 1 School of Mechanical Engineering, Pusan National University, Busan 46241, Korea; jppark@pusan.ac.kr; 2 Department of Industrial Engineering, Pusan National University, Busan 46241, Korea; cp@pusan.ac.kr; 3 Department of Physics, Myongji University, Yongin 17058, Korea; jwcho@mju.ac.kr

**Keywords:** crack initiation test, estimation uncertainty, Weibull distribution, time dependent, interval censored, Monte Carlo simulation

## Abstract

It is extremely difficult to predict the initiation time of cracking due to a large time spread in most cracking experiments. Thus, probabilistic models, such as the Weibull distribution, are usually employed to model the initiation time of cracking. Therefore, the parameters of the Weibull distribution are estimated from data collected from a cracking test. However, although the development of a reliable cracking model under ideal experimental conditions (e.g., a large number of specimens and narrow censoring intervals) could be achieved in principle, it is not straightforward to quantitatively assess the effects of the ideal experimental conditions on model estimation uncertainty. The present study investigated the effects of key experimental conditions, including the time-dependent effect of the censoring interval length, on the estimation uncertainties of the Weibull parameters through Monte Carlo simulations. The simulation results provided quantified estimation uncertainties of Weibull parameters in various cracking test conditions. Hence, it is expected that the results of this study can offer some insight for experimenters developing a probabilistic crack initiation model by performing experiments.

## 1. Introduction

It is widely known that stress corrosion cracking (SCC) can result in loss-of-coolant accidents in nuclear reactors [1,2,3]. Thus, the prediction of the SCC initiation time is a very important task for several researchers in nuclear science. However, this is a difficult task due to the complex mechanism of SCC initiation, which is not clearly identified yet. Therefore, empirical SCC initiation models are generally adopted for this purpose [4,5,6].

However, most SCC experiments showed non-negligible scatter with respect to cracking time [7], although all of the experimental conditions (e.g., temperature, tensile stress, etc.) were strictly controlled. Therefore, a probabilistic model was frequently used as an SCC initiation model to quantitatively consider the time scatter. Particularly, the Weibull distribution [8], which can generally consider the effect of the time-dependent degradation of a material, is widely accepted as a probabilistic model of SCC initiation time [6,9,10].

To obtain the model parameters of SCC initiation (i.e., Weibull parameters in this case), a cracking test must be performed. The typical procedure of a cracking test involves an interval-censored reliability test. This implies that several stressed specimens (e.g., U-bend and constant tensile stress specimens) are exposed to a corrosive environment and censored at every scheduled time. Following the test, the testing results can be used to estimate the Weibull parameters typically using either the maximum likelihood estimation (MLE) or the median rank regression (MRR) method [11].

Both of the aforementioned methods produce different estimates from the same SCC testing data [12]. However, it is expected that the reliability of the Weibull estimators increases with an increase in the number of test specimens and a smaller length of the censoring interval (LCI). However, it is not possible to study the quantitative effect by idealizing these experimental conditions (i.e., the effect of increasing the number of specimens and narrowing LCI, etc.), because there is no theory yet available to calculate the exact estimation uncertainties for Weibull parameters with interval-censored data [11].

Therefore, in our previous study [12], the effects of certain experimental conditions on estimation uncertainties of Weibull parameters were investigated through the Monte Carlo simulation. However, the study also revealed that the following issues warranted further investigation:

- In the previous study, it was assumed that LCI was a time-independent variable. However, time-dependent LCI is more general for real SCC tests. Thus, it is necessary to investigate the effect of time dependence on LCI.
- In the previous study, it was not appropriate to use the test duration as an experimental factor of estimation uncertainty because it is not possible for experimenters to know the relative test duration (RTD). Additionally, it was possible to continue the simulation even after all of the specimens cracked if the test duration was used as a fixed input for the simulation study [12].

This study examines the above issues by comparing a time-dependent LCI (TDLCI) scheme with a time-independent LCI (TILCI) scheme and by adopting the end cracking fraction (ECF) instead of the test duration as an experimental factor of estimation uncertainty.

## 2. Theoretical Background of the Weibull Distribution

In this section, we will discuss the reason for the suitability of the Weibull distribution as an SCC initiation model.

### 2.1. Extremal Types Theorem

According to the well-known central limit theorem [13], the distribution of an independent and identically distributed (iid) sample mean can be approximated to the normal distribution with a large sample. A similar theorem known as the extremal types theorem exists for the distribution of sample maxima (or minima). The extremal types theorem [14,15] states that the location scale families of only three distributions are possible limits for the distribution of sample maxima (or minima).

**Figure 1:**
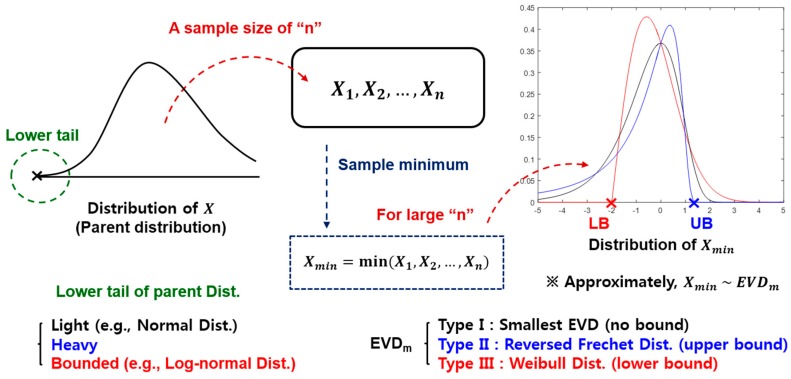
Schematic illustration of the extremal types theorem for sample minima. EVD, extreme value distribution.

Figure 1 shows the schematic illustration of the extremal types theorem for sample minima. Three possible cases for the distribution of sample minima include the following:

- If the unbounded lower tail of a parent distribution is light-tailed (i.e., falls off exponentially or faster [16]), then the distribution of sample minima is approximated to a Type I extreme value distribution for minima (Type I EVD_m_, i.e., the smallest extreme value distribution) that has an unbounded domain.
- the unbounded lower tail of the parent distribution is heavy-tailed, then the distribution of the sample minima is approximated to a Type II EVD_m_ (i.e., reversed Fréchet distribution) that has an upper bound.
- the lower tail of the parent distribution is bounded (e.g., uniform distribution), then the distribution of the sample minima is approximated to a Type III EVD_m_ (i.e., Weibull distribution) that has a lower bound.

### 2.2. Weibull Distribution

McFadden [17] proved that all three types of EVD could be expressed as a one functional form, namely the generalized extreme value distribution (GEVD). The probability density function (PDF) of GEVD for the sample minima case could be expressed as follows:

- Type I EVD_m_: smallest extreme value distribution (ξ=0):
  (1) $$g\left(x;\mu ,\sigma ,0\right)=\left(\frac{1}{\sigma }\right)exp\left[-exp\left(\frac{x+\mu }{\sigma }\right)+\left(\frac{x+\mu }{\sigma }\right)\right],\;x\in \mathbb{R},$$
- Type II EVD_m_: reversed Fréchet distribution (ξ>0):
  (2) $$g\left(x;\mu ,\sigma ,\xi \right)=\left(\frac{1}{\sigma }\right)exp\left[-{\left(1-\xi \left(\frac{x+\mu }{\sigma }\right)\right)}^{-\frac{1}{\xi }}\right]{\left[1-\xi \left(\frac{x+\mu }{\sigma }\right)\right]}^{-\frac{1}{\xi }-1},\;x\leq \mu -\frac{\sigma }{\xi },$$
- Type III EVD_m_: Weibull distribution (ξ<0):
  (3) $$g\left(x;\mu ,\sigma ,\xi \right)=\left(\frac{1}{\sigma }\right)exp\left[-{\left(1-\xi \left(\frac{x+\mu }{\sigma }\right)\right)}^{-\frac{1}{\xi }}\right]{\left[1-\xi \left(\frac{x+\mu }{\sigma }\right)\right]}^{-\frac{1}{\xi }-1},\;x\geq \mu -\frac{\sigma }{\xi },$$
  where x denotes the variable, μ∈ℝ denotes the location parameter, σ>0 denotes the scale parameter and ξ∈ℝ denotes the shape parameter. The type of EVD is determined by the sign of ξ.

Specifically, the Type III EVD_m_ in Equation (3) can be converted to a widely-known functional form of the Weibull distribution by parameter substitution as given by:

(4) $$x=t-2\eta ,\mu =\eta ,\sigma =\frac{\eta }{\beta },\xi =-\frac{1}{\beta }.$$

Following the substitution, a two-parameter Weibull distribution that is frequently used as a cracking probability model is obtained with the following cumulative distribution function (CDF):

(5) $$F\left(t;\beta ,\eta \right)=1-exp\left[-{\left(\frac{t}{\eta }\right)}^{\beta }\right],\;$$

where t≥0 denotes time, β>0 denotes the shape parameter and η>0 denotes the scale parameter of the Weibull distribution.

In applications of the cracking test, the cracking time of a specimen indicates the earliest cracking time of the specimen (i.e., the minimum cracking time). Therefore, the distribution of cracking time at a macroscopic scale (e.g., at engineering scale) will follow the EVD_m_ irrespective of the distribution of cracking time at a microscopic scale (e.g., at a grain boundary scale). Furthermore, it is evidently not possible for the cracking time to correspond to a negative value. That is, the lower tail of the parent distribution (i.e., distribution of cracking time at a microscopic scale) is bounded. Therefore, the distribution of cracking time at a macroscopic scale corresponds to the Type III EVD_m_ (i.e., Weibull distribution).

Furthermore, it is interesting that, when iid Weibull distributed samples (e.g., cracking time) are selected, then the distribution of the sample minima also follows the Weibull distribution [11]. Thus, if the cracking mechanism is governed by the weakest link behavior [11], then this fact could be a strong basis to justify the use of the Weibull distribution as an appropriate statistical model of crack initiation time.

### 2.3. Estimation of Weibull Parameters

As previously mentioned, two methods are usually used to estimate the Weibull parameters from the given test data. These include median rank regression (MRR) and maximum likelihood estimation (MLE) [11,12].

The MRR method uses cracking fraction data at each censoring time. It is possible to calculate the cracking probability at each censoring time by the median rank concept based on the assumption that all of the specimens were tested independently [11,12]. Thus, the unreliability function (i.e., Weibull distribution) can be finally obtained by regression. In contrast, MLE uses the cracking time information of each specimen. Weibull estimators can be obtained by finding the point at which the likelihood function is maximized. The detailed procedures of MRR and MLE were well described in a previous study [12]. The present study uses the same procedure for Weibull estimation. The estimates were numerically calculated by the MATLAB (R2015b, MathWorks, Natick, MA, USA, 2015) function fsolve (for MLE) and lsqcurvefit (for MRR) [12], although there are other statistical software packages available, where the Weibull estimation can also be performed.

An interesting point is that the Weibull estimates calculated by MRR or MLE are different from each other even though both of the estimates were derived from the same test data [12]. Thus, it is also important to examine the reliability of each estimator at a given experimental condition.

## 3. Monte Carlo Simulation

### 3.1. Experimental Factors

Experimental factors (e.g., number of specimens and LCI) and the method of estimation (e.g., MRR and MLE) can affect the uncertainties of Weibull estimators. In the present study, a Monte Carlo simulation was performed to investigate their corresponding quantitative effects. The experimental factors considered in the simulation study include (1) true Weibull parameters; (2) the number of specimens; (3) end cracking fractions; (4) starting LCI; and (5) the time dependence of LCI.

#### 3.1.1. True Weibull Parameters

As previously mentioned, it could be reasonably assumed that the inherent cracking probability was Weibull distributed at a macroscopic scale. The study investigated as to whether the estimation uncertainties were affected by the parameters of the given Weibull distribution (i.e., inherent cracking probability behavior). These were termed as the true Weibull parameters ($\beta_{\mathrm{true}},{\;\eta }_{\mathrm{true}}$), and they are generally unknown to experimenters.

It should be noted that the scale parameter η was considered as a nuisance parameter in several applications [18]. For example, if the relative errors (RE) of estimators that were defined as follows:

(6) $$\mathrm{RE}\left(\hat{\beta }\right)=\;\frac{\hat{\beta }-\beta_{\mathrm{true}}}{\beta_{\mathrm{true}}};\;\mathrm{RE}\left(\hat{\eta }\right)=\;\frac{\hat{\eta }-\eta_{\mathrm{true}}}{\eta_{\mathrm{true}}},$$

were affected by the value of the true scale parameter ($\eta_{\mathrm{true}}$), then only changing the time unit (e.g., hours to seconds) could affect the relative estimation errors. This is contradictory. Therefore, the $\eta_{\mathrm{true}}$ is just a scale factor, and relative estimation errors are not affected by the value of $\eta_{\mathrm{true}}$ [19]. Without loss of generality, the value of $\eta_{\mathrm{true}}$ could be fixed at 100 in this simulation study.

Nevertheless, the value of the true Weibull shape parameter ($\beta_{\mathrm{true}}$) could be a factor affecting the relative estimation errors. That is, the shape parameter was a main parameter of the Weibull distribution [18]. In order to examine this effect, several values of $\beta_{\mathrm{true}}$ (2, 3 and 4) were selected as the simulation inputs. In previous studies, the value of the estimated Weibull shape parameter for an SCC initiation time ranged from two to four [6,20,21,22].

#### 3.1.2. Number of Specimens

It was expected that the estimated Weibull parameters (i.e., $\hat{\beta },\;\hat{\eta }$) could be reliable with a large number of specimens. However, the SCC initiation test for nuclear reactor materials requires a corrosive environment with high temperatures and pressures. Thus, it is difficult to test a large number of specimens simultaneously. Hence, the simulation range of the specimen number was set from five to 50.

#### 3.1.3. End Cracking Fraction

During the performance of the SCC test, cracking does not necessarily occur for every specimen within the available testing time. Thus, the test duration was considered as a factor of estimation uncertainties in an earlier study [12]. However, the results indicated that there were deficiencies to using the test duration as a factor of estimation uncertainties. First, experimenters did not know their relative test duration (RTD), which is defined as follows:

(7) $$\mathrm{RTD}=\frac{test\;duration}{\eta_{\mathrm{true}}},$$

this is because the experimenters did not know the exact value of $\eta_{\mathrm{true}}$. Additionally, it was possible to continue the simulation even after all of the specimens cracked when the test duration was used as a fixed input for a simulation study [12]. This is an undesirable phenomenon for the MRR estimation because it produces redundant and often irrelevant results close to the end time of the simulation.

Therefore, the end cracking fraction (ECF) is considered as an alternative factor of estimation uncertainties. For example, if the value of ECF corresponded to 0.6, then the test ended when more than or equal to 60% of the specimens cracked. The simulation range for ECF was set from 0.6 to 1.0.

#### 3.1.4. Length of Censoring Interval

In most cases, it is expected that a shorter LCI is better to estimate reliable Weibull parameters. However, frequent censoring causes inconveniences for experimenters. Thus, it is important to set a reasonable LCI for a cracking test. In order to investigate the general effect of LCI, the simulation range for starting LCI was set from 5% to 50% of $\eta_{\mathrm{true}}$, although the value of $\eta_{\mathrm{true}}$ was unknown in the real testing case.

As previously mentioned, the value of $\beta_{\mathrm{true}}$ exceeded unity for the SCC initiation of nuclear materials [6,20,21,22]. That is, the hazard function of the true Weibull distribution increased with time. Therefore, it was reasonable to narrow the LCI with respect to time. However, it was quite difficult for experimenters to determine an appropriate censoring scheme because it was not possible for the experimenter to know the hazard function of the true Weibull distribution for the given SCC initiation case.

**Figure 2:**
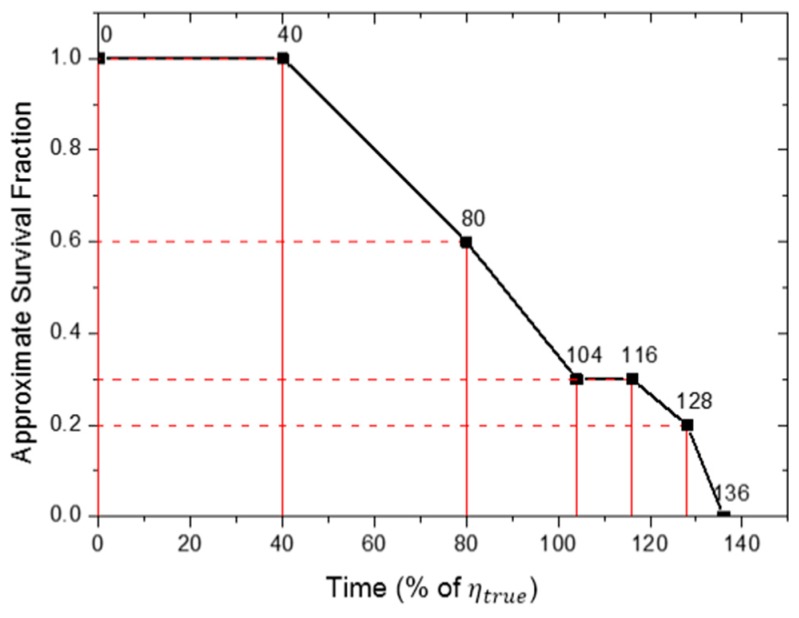
Relation between the approximate surviving fraction and censoring time for the simulation experiment with the time-dependent LCI (TDLCI) scheme (data from Table 1).

Therefore, in this study, the simple time-dependent LCI (TDLCI) scheme was used in which the LCI varied based on the surviving fraction of test specimens. Table 1 shows an example of a set of data generated by a random simulation when the TDLCI scheme was applied. As observed, the LCI decreased with decreases in the surviving fractions of specimens. This tendency is graphically represented in Figure 2, which plots the relation between the approximate surviving fraction and testing time from the data in Table 1.

**Figure 3:**
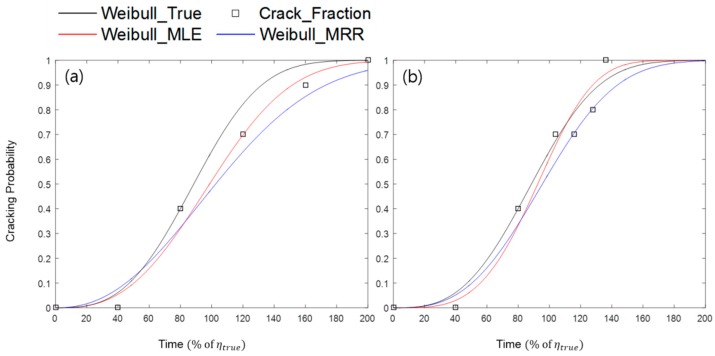
Two examples of the random simulation experiment with (**a**) the time-independent LCI (TILCI) scheme and (**b**) the TDLCI scheme (number of specimens: 10; starting LCI: 40% of $\eta_{\mathrm{true}}$; ECF: 1.0; $\beta_{\mathrm{true}}$: 3.0). MRR: median rank regression, MLE: maximum likelihood estimation.

As a control group, time-independent LCI (TILCI) cases were also studied. Figure 3 shows the comparison of two random simulation examples with the same experimental factors except for the LCI scheme (i.e., TILCI vs. TDLCI). Specifically, the points in Figure 3b exactly corresponded to the data in Table 1. The black line denotes the pre-assumed true Weibull distribution that indicated the real cracking probability; the black dots denote randomly-generated cracking data from the pre-assumed true Weibull distribution; the red line denotes the estimated Weibull distribution from the cracking data by the MLE method; and the blue line denotes the estimated Weibull distribution from the cracking data by the MRR method. In this case, the results indicated that the estimated Weibull curves were much closer to their true Weibull distribution when the TDLCI scheme was applied for the test. However, this tendency was not always valid for all random simulations.

### 3.2. Simulation Approach

**Figure 4:**
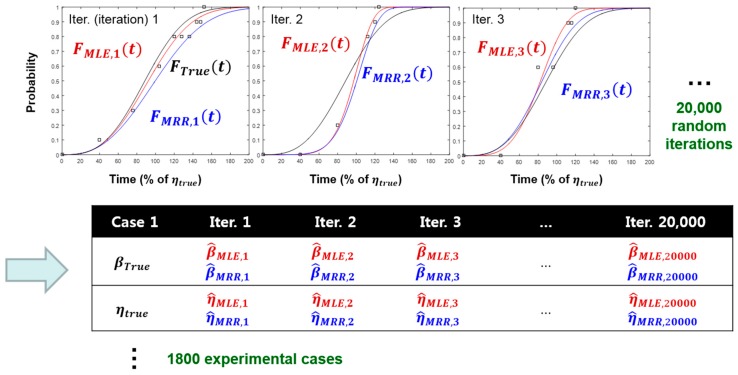
Schematic procedure of the Monte Carlo simulation study.

Table 2 shows the simulation range of the study. A total of 1800 (=1 × 3 × 10 × 3 × 10 × 2) experimental cases were considered, and 20,000 random iterations were performed for each experimental case. From the resulting simulation data, the Weibull estimators (i.e., $\hat{\beta }$, $\hat{\eta }$) were calculated by both the MLE and MRR methods. Figure 4 shows the schematic procedure of the simulation study.

**Figure 5:**
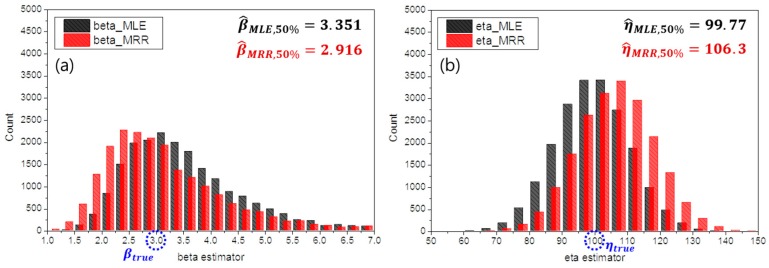
Distributions of 20,000 replicates of parameter estimates with respect to the experimental conditions in Table 3 for (**a**) β and (**b**) η (red bars: estimates by the MLE method; black bars: estimates by the MRR method).

Following the simulation, the distributions of $\hat{\beta }$ and $\hat{\eta }$ for each experimental case were obtained. For example, Figure 5 shows the distribution of estimates when the experimental factors were given as shown in Table 3. As shown in Figure 5, the dispersion and bias of the estimates could be changed by the method of estimation, as well as a combination of experimental factors. In this case, the MRR method exhibited a smaller bias for $\hat{\beta }$ when compared to that of the MLE. In contrast, the MLE displayed a smaller bias for $\hat{\eta }$.

## 4. Results and Discussion

### 4.1. Mean Number of Censoring

If a value of ECF was fixed as an input factor for a simulation experiment, then the number of censoring times during the simulation experiment corresponded to a random variable. Therefore, it was possible to simply calculate the mean number of censoring (MNC) for 20,000 replicates of each experimental case. Let i denote the i-th replicate for a given experimental case, then the MNC for that experimental case can be defined as follows:

(8) $$\mathrm{MNC}=\frac{\sum_{i=1}^{20,000}\left(Number\;of\;censoring\;times\;for\;the\;ith\;replicate\right)}{Number\;of\;total\;replicates\left(=20,000\right)}.$$

**Figure 6:**
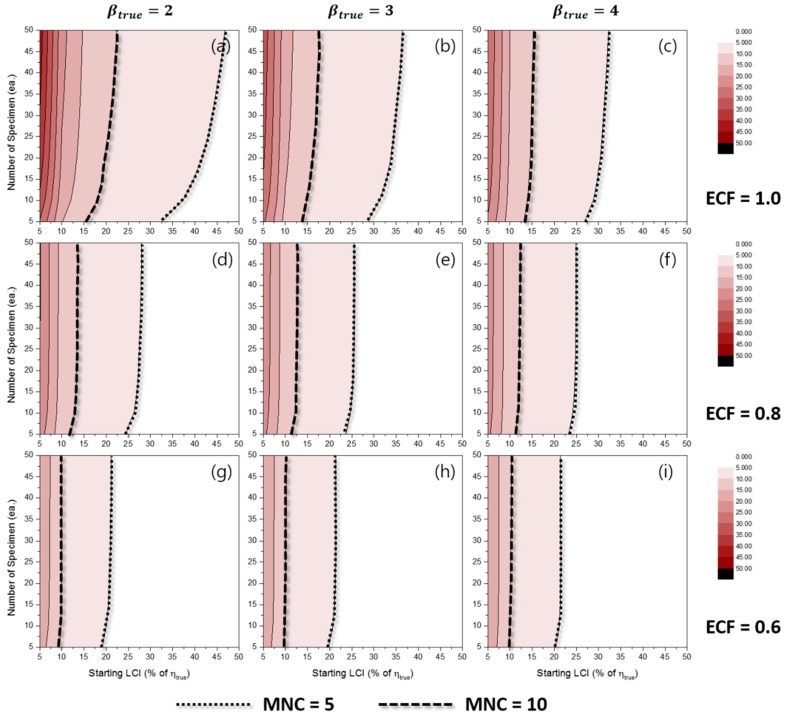
Effects of the number of specimens and starting LCI on the mean number of censoring (MNC) for the TILCI case when ECF = 1.0 and (**a**) $\beta_{\mathrm{true}}=2$, (**b**) $\beta_{\mathrm{true}}=3$, (**c**) $\beta_{\mathrm{true}}=4$; when ECF = 0.8 and (**d**) $\beta_{\mathrm{true}}=2$, (**e**) $\beta_{\mathrm{true}}=3$, (**f**) $\beta_{\mathrm{true}}=4$; when ECF = 0.6 and (**g**) $\beta_{\mathrm{true}}=2$, (**h**) $\beta_{\mathrm{true}}=3$, (**i**) $\beta_{\mathrm{true}}=4$.

**Figure 7:**
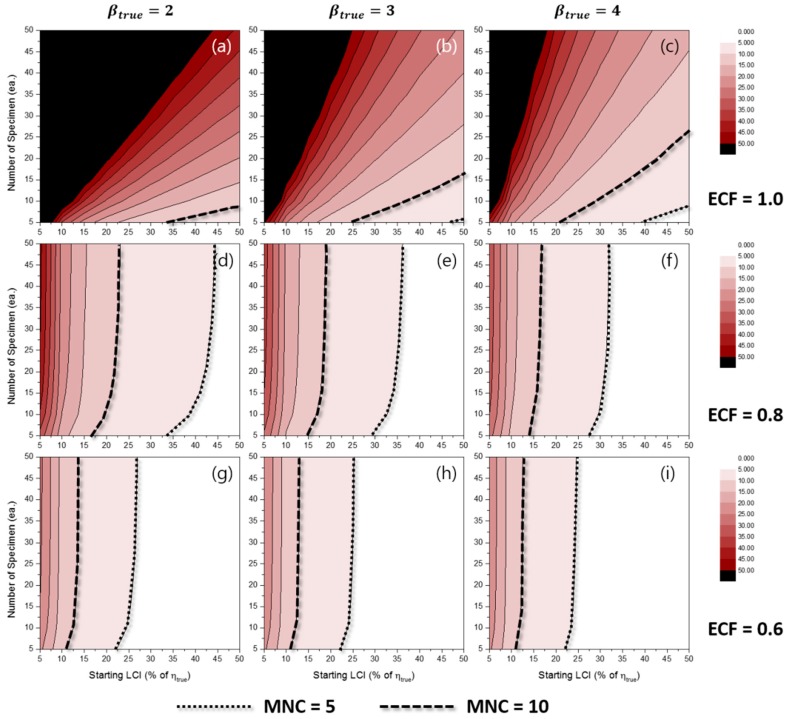
Effects of the number of specimens and starting LCI on MNC for the TDLCI case when ECF = 1.0 and (**a**) $\beta_{\mathrm{true}}=2$, (**b**) $\beta_{\mathrm{true}}=3$, (**c**) $\beta_{\mathrm{true}}=4$; when ECF = 0.8 and (**d**) $\beta_{\mathrm{true}}=2$, (**e**) $\beta_{\mathrm{true}}=3$, (**f**) $\beta_{\mathrm{true}}=4$; when ECF = 0.6 and (**g**) $\beta_{\mathrm{true}}=2$, (**h**) $\beta_{\mathrm{true}}=3$, (**i**) $\beta_{\mathrm{true}}=4$.

Figure 6 represents the effects of some experimental conditions for MNC when the TILCI scheme is applied, and Figure 7 represents the effects of some experimental conditions for MNC when the TDLCI scheme is applied.

A general tendency of increasing MNC was observed with: (1) a large number of specimens; (2) a narrow starting LCI; (3) a high value of ECF; (4) a low value of $\beta_{\mathrm{true}}$; and (5) the application of the TDLCI scheme. It is shown that if all other experimental factors are the same (e.g., the number of specimens and starting LCI), the TDLCI case requires much more MNC when compared to that of the TILCI case. This is an unwanted phenomenon for experimenters who want to insist on the efficiency of the TDLCI scheme. Therefore, the same MNC line was set as a criterion of uncertainty comparison between the TILCI and TDLCI schemes. In Figure 6 and Figure 7, the black dotted line denotes MNC = 5, and the black dashed line denotes MNC = 10.

### 4.2. Mean Test Duration 

If a value of ECF is fixed, then the test duration also becomes a random variable. Thus, it was possible to simply calculate the mean test duration (MTD) of 20,000 replicates for each experimental case. Given that i is an index of random simulation for a certain experimental case, then the MTD can be expressed as follows:

(9) $$\mathrm{MTD}=\frac{\sum_{i=1}^{20,000}\left(RTD\;for\;ith\;random\;simulation\right)}{Number\;of\;replicates\left(=20,000\right)}.$$

**Figure 8:**
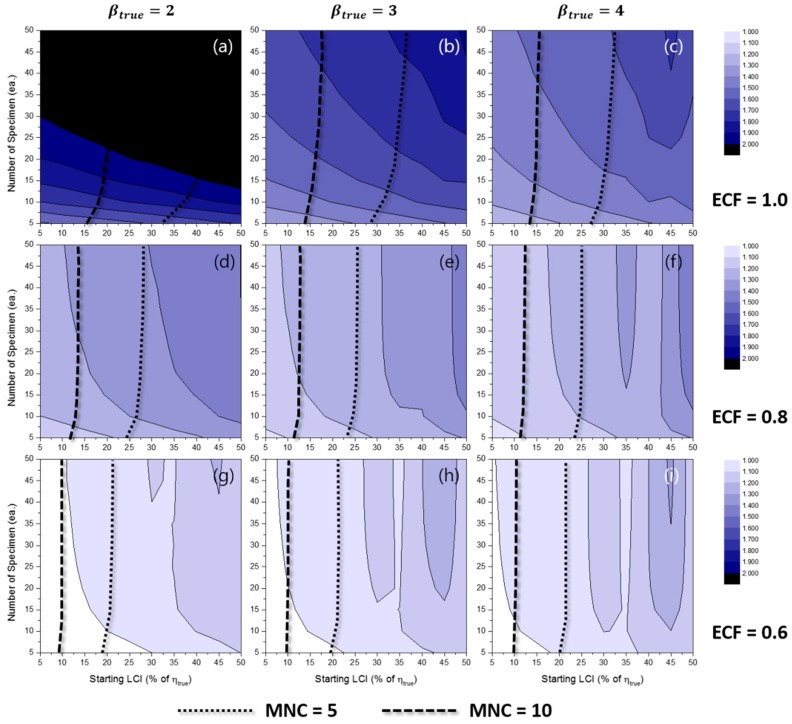
Effects of the number of specimens and starting LCI on the mean test duration (MTD) for the TILCI case when ECF = 1.0 and (**a**) $\beta_{\mathrm{true}}=2$, (**b**) $\beta_{\mathrm{true}}=3$, (**c**) $\beta_{\mathrm{true}}=4$; when ECF = 0.8 and (**d**) $\beta_{\mathrm{true}}=2$, (**e**) $\beta_{\mathrm{true}}=3$, (**f**) $\beta_{\mathrm{true}}=4$; when ECF = 0.6 and (**g**) $\beta_{\mathrm{true}}=2$, (**h**) $\beta_{\mathrm{true}}=3$, (**i**) $\beta_{\mathrm{true}}=4$.

**Figure 9:**
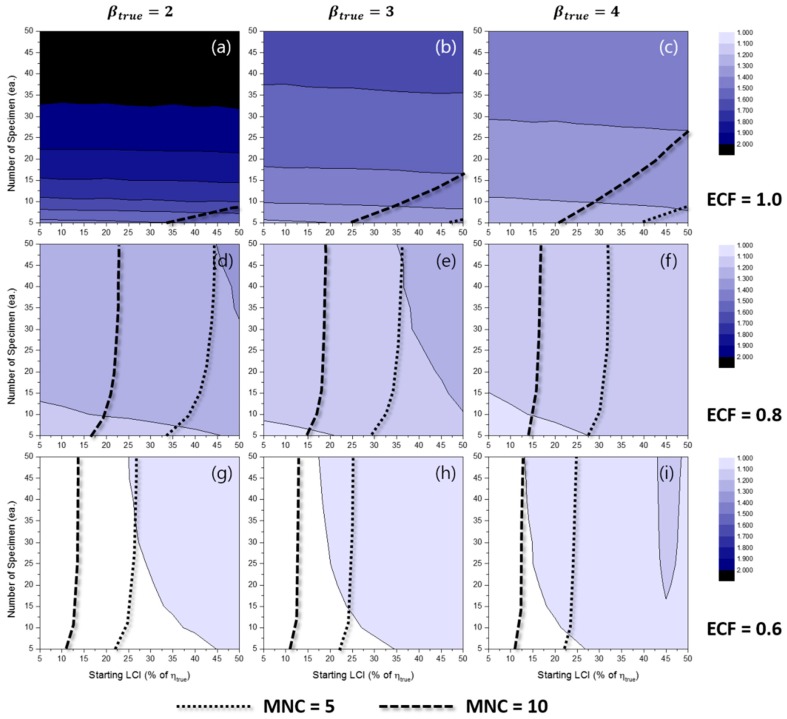
Effects of the number of specimens and starting LCI on MTD for the TDLCI case when ECF = 1.0 and (**a**) $\beta_{\mathrm{true}}=2$, (**b**) $\beta_{\mathrm{true}}=3$, (**c**) $\beta_{\mathrm{true}}=4$; when ECF = 0.8 and (**d**) $\beta_{\mathrm{true}}=2$, (**e**) $\beta_{\mathrm{true}}=3$, (**f**) $\beta_{\mathrm{true}}=4$; when ECF = 0.6 and (**g**) $\beta_{\mathrm{true}}=2$, (**h**) $\beta_{\mathrm{true}}=3$, (**i**) $\beta_{\mathrm{true}}=4$.

Figure 8 represents the effects of experimental conditions for MTD when the TILCI scheme is applied, and Figure 9 represents the effects of experimental conditions for MTD when the TDLCI scheme is applied. A tendency of increasing MTD was observed with: (1) a large number of specimens; (2) a wide starting LCI; (3) a high value of ECF; and (4) the application of the TILCI scheme. However, with respect to the case when the value of ECF was relatively low, there was a non-consistent effect of starting LCI for MTD (see Figure 8i). This unusual effect will be examined in a future study.

The results indicated that a low value of $\beta_{\mathrm{true}}$ resulted in increasing MTD when ECF ≥ 0.8 and decreasing MTD when ECF corresponded to 0.6. It was suspected that the branch point of ECF would correspond to 0.632 (i.e., F(t=η)) because the CDFs of the Weibull distribution possessed different β crosses at the point (η, F(η)).

**Figure 10:**
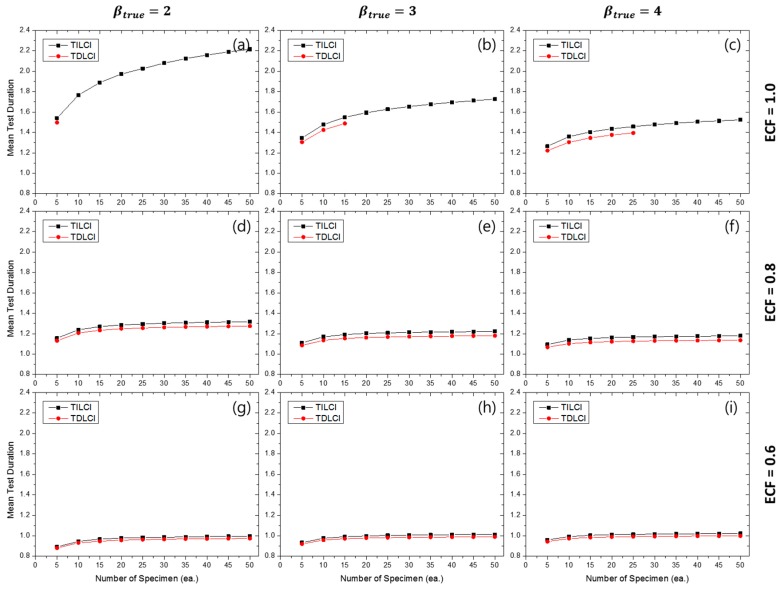
Effects of the number of specimens on MTD for MNC = 10 lines when ECF = 1.0 and (**a**) $\beta_{\mathrm{true}}=2$, (**b**) $\beta_{\mathrm{true}}=3$, (**c**) $\beta_{\mathrm{true}}=4$; when ECF = 0.8 and (**d**) $\beta_{\mathrm{true}}=2$, (**e**) $\beta_{\mathrm{true}}=3$, (**f**) $\beta_{\mathrm{true}}=4$; when ECF = 0.6 and (**g**) $\beta_{\mathrm{true}}=2$, (**h**) $\beta_{\mathrm{true}}=3$, (**i**) $\beta_{\mathrm{true}}=4$.

In Figure 10, it was observed that the TDLCI cases corresponded to a slightly shorter MTD when compared with the TILCI case given that the same MNC line is applied as a criterion. However, there is a very small difference between the two schemes with respect to MTD.

### 4.3. Empirical Confidence Interval of $\hat{\beta }$

From the random simulation result, the 5th, 50th and 95th percentiles (${\hat{\beta }}_{5\%},{\hat{\beta }}_{50\%},\;{\hat{\beta }}_{95\%};{\hat{\eta }}_{5\%},{\hat{\eta }}_{50\%},\;{\hat{\eta }}_{95\%}$) of 20,000 replicates of Weibull estimates could be derived for each experimental case. The median estimates (i.e., ${\hat{\beta }}_{50\%},\;{\hat{\eta }}_{50\%}$) could be selected from these estimates and converted to the relative error for median estimate (${\mathrm{RE}}_{50\%}$) to represent the bias of estimators, which is defined as follows:

(10) $${\mathrm{RE}}_{50\%}\left(\hat{\beta }\right)=\mathrm{RE}\left({\hat{\beta }}_{50\%}\right)=\frac{{\hat{\beta }}_{50\%}-\beta_{\mathrm{true}}}{\beta_{\mathrm{true}}};\;{\mathrm{RE}}_{50\%}\left(\hat{\eta }\right)=\mathrm{RE}\left({\hat{\eta }}_{50\%}\right)=\frac{{\hat{\eta }}_{50\%}-\eta_{\mathrm{true}}}{\eta_{\mathrm{true}}},$$

and in order to quantify the dispersion of estimators, a relative length of a 90% confidence interval (${\mathrm{RLCI}}_{90\%}$) was utilized, which is defined as follows:

(11) $${\mathrm{RLCI}}_{90\%}\left(\hat{\beta }\right)=\mathrm{RE}\left({\hat{\beta }}_{95\%}\right)-\mathrm{RE}\left({\hat{\beta }}_{5\%}\right);{\;\mathrm{RLCI}}_{90\%}\left(\hat{\eta }\right)=\mathrm{RE}\left({\hat{\eta }}_{95\%}\right)-\mathrm{RE}\left({\hat{\eta }}_{5\%}\right).$$

**Figure 11:**
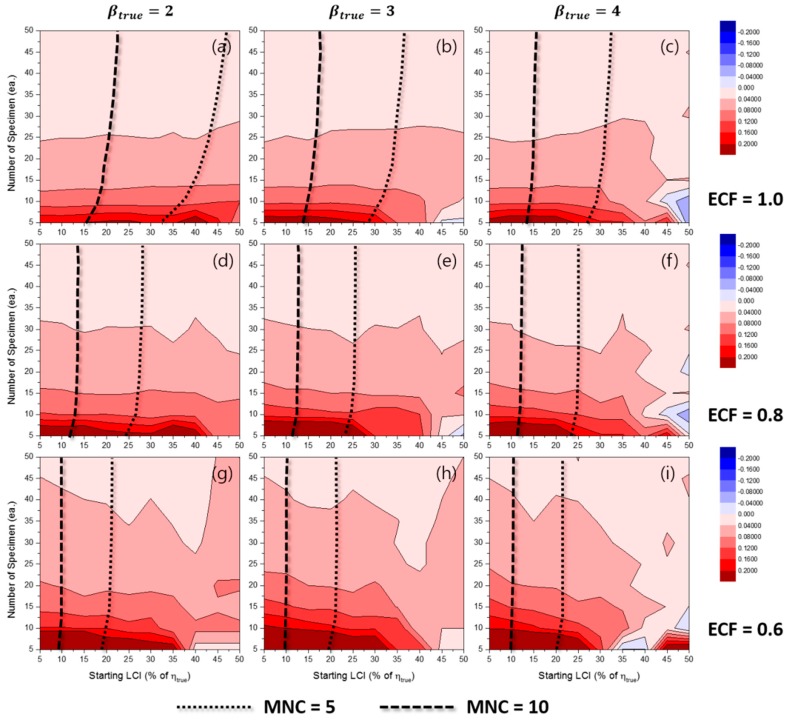
Effects of the number of specimens and starting LCI on ${\mathrm{RE}}_{50\%}\left({\hat{\beta }}_{\mathrm{MLE}}\right)$ for the TILCI case when ECF = 1.0 and (**a**) $\beta_{\mathrm{true}}=2$, (**b**) $\beta_{\mathrm{true}}=3$, (**c**) $\beta_{\mathrm{true}}=4$; when ECF = 0.8 and (**d**) $\beta_{\mathrm{true}}=2$, (**e**) $\beta_{\mathrm{true}}=3$, (**f**) $\beta_{\mathrm{true}}=4$; when ECF = 0.6 and (**g**) $\beta_{\mathrm{true}}=2$, (**h**) $\beta_{\mathrm{true}}=3$, (**i**) $\beta_{\mathrm{true}}=4$.

Figure 11 shows the contour plots of ${\mathrm{RE}}_{50\%}\left({\hat{\beta }}_{\mathrm{MLE}}\right)$ for the TILCI case, and this indicated a relative estimation bias in the Weibull shape parameter with respect to the MLE method. It was likely that ${\hat{\beta }}_{\mathrm{MLE}}$ showed a tendency to be overestimated irrespective of the value of ECF and $\beta_{\mathrm{true}}$ when the number of specimens was relatively small. The unusual result that occurred in the long starting LCI region may not be reliable due to the low convergence ratio (as shown in Figure S1 in the Supplementary Materials).

**Figure 12:**
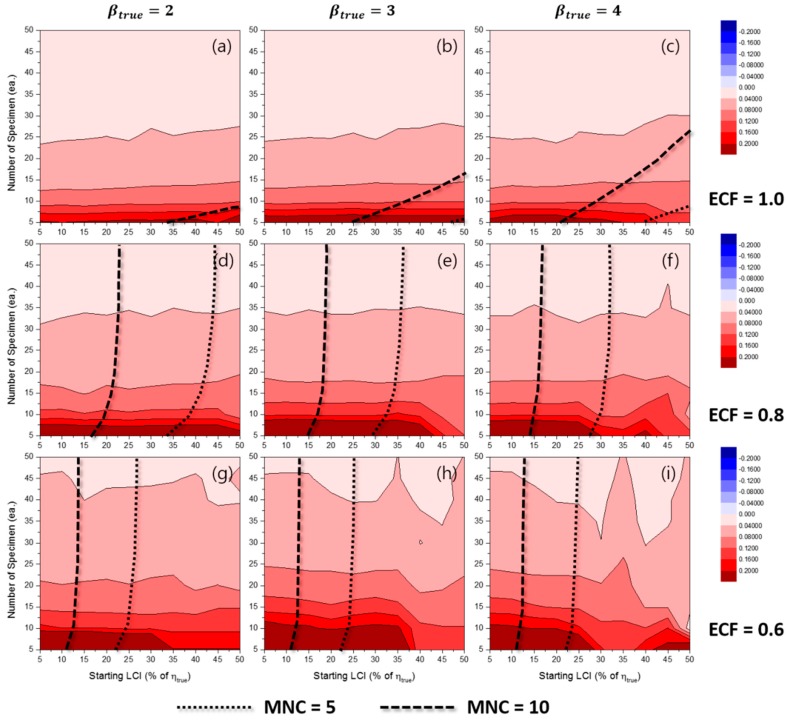
Effects of the number of specimens and starting LCI on ${\mathrm{RE}}_{50\%}\left({\hat{\beta }}_{\mathrm{MLE}}\right)$ for the TDLCI case when ECF = 1.0 and (**a**) $\beta_{\mathrm{true}}=2$, (**b**) $\beta_{\mathrm{true}}=3$, (**c**) $\beta_{\mathrm{true}}=4$; when ECF = 0.8 and (**d**) $\beta_{\mathrm{true}}=2$, (**e**) $\beta_{\mathrm{true}}=3$, (**f**) $\beta_{\mathrm{true}}=4$; when ECF = 0.6 and (**g**) $\beta_{\mathrm{true}}=2$, (**h**) $\beta_{\mathrm{true}}=3$, (**i**) $\beta_{\mathrm{true}}=4$.

Figure 12 shows the ${\mathrm{RE}}_{50\%}\left({\hat{\beta }}_{\mathrm{MLE}}\right)$ for the TDLCI case. As shown in both Figure 11 and Figure 12, the effect of starting LCI on ${\mathrm{RE}}_{50\%}\left({\hat{\beta }}_{\mathrm{MLE}}\right)$ was very little when the starting LCI was relatively short and the value of ECF was high. It is shown that the overall convergence ratio was increased with the TDLCI scheme (as shown in Figure S2). However, the convergence ratios for the TILCI and TDLCI case along the same MNC line were very similar (as shown in Figure S3).

With respect to the case of MRR estimators, the important difference between MLE and MRR was that the convergence ratio for MRR estimation almost approached unity in every experimental combination (as shown in the Supplementary Materials). Thus, it could be concluded that there was no filtering effect [12] for the MRR cases.

In a manner similar to the ${\hat{\beta }}_{\mathrm{MLE}}$ case, it was likely that ${\hat{\beta }}_{\mathrm{MRR}}$ tended to be overestimated (as shown in Figures S4 and S5) when the number of specimens was relatively small although there were some exceptions (as shown in Figure S5a–c). When the value of ECF is relatively low, a strong overestimation tendency for ${\hat{\beta }}_{\mathrm{MRR}}$ was observed in both the TILCI and TDLCI cases.

From the simulation result, it was difficult to find the general effect of experimental factors on ${\mathrm{RE}}_{50\%}\left({\hat{\beta }}_{\mathrm{MRR}}\right)$ and also on ${\mathrm{RE}}_{50\%}\left({\hat{\beta }}_{\mathrm{MLE}}\right)$, except for the number of specimens. For example, in the case of ECF = 0.6, $\beta_{\mathrm{true}}=4$ and starting LCI at 40% of $\eta_{\mathrm{true}}$, a strange valley at which a rapidly decreased bias of ${\hat{\beta }}_{\mathrm{MRR}}$ (as shown in Figures S4i and S5i) was observed.

**Figure 13:**
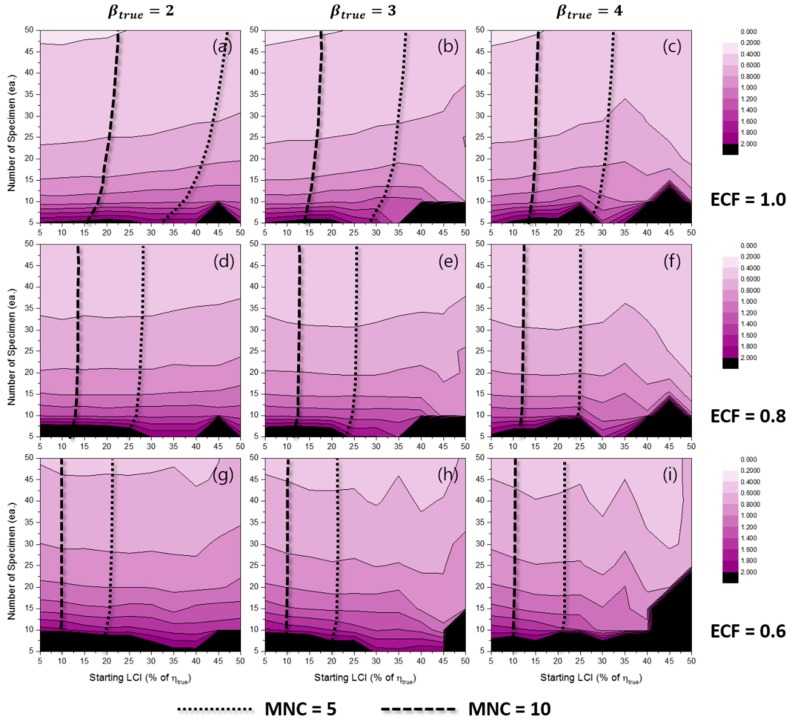
Effects of the number of specimens and starting LCI on ${\mathrm{RLCI}}_{90\%}\left({\hat{\beta }}_{\mathrm{MLE}}\right)$ for the TILCI case when ECF = 1.0 and (**a**) $\beta_{\mathrm{true}}=2$, (**b**) $\beta_{\mathrm{true}}=3$, (**c**) $\beta_{\mathrm{true}}=4$; when ECF = 0.8 and (**d**) $\beta_{\mathrm{true}}=2$, (**e**) $\beta_{\mathrm{true}}=3$, (**f**) $\beta_{\mathrm{true}}=4$; when ECF = 0.6 and (**g**) $\beta_{\mathrm{true}}=2$, (**h**) $\beta_{\mathrm{true}}=3$, (**i**) $\beta_{\mathrm{true}}=4$.

**Figure 14:**
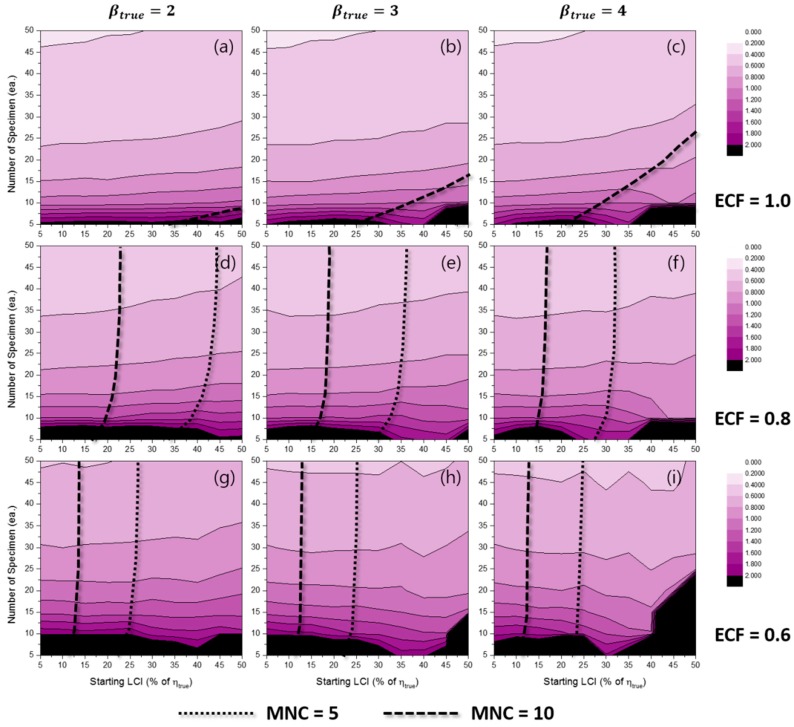
Effects of the number of specimens and starting LCI on ${\mathrm{RLCI}}_{90\%}\left({\hat{\beta }}_{\mathrm{MLE}}\right)$ for the TDLCI case when ECF = 1.0 and (**a**) $\beta_{\mathrm{true}}=2$, (**b**) $\beta_{\mathrm{true}}=3$, (**c**) $\beta_{\mathrm{true}}=4$; when ECF = 0.8 and (**d**) $\beta_{\mathrm{true}}=2$, (**e**) $\beta_{\mathrm{true}}=3$, (**f**) $\beta_{\mathrm{true}}=4$; when ECF = 0.6 and (**g**) $\beta_{\mathrm{true}}=2$, (**h**) $\beta_{\mathrm{true}}=3$, (**i**) $\beta_{\mathrm{true}}=4$.

Figure 13 and Figure 14 show the contour plots of ${\mathrm{RLCI}}_{90\%}\left({\hat{\beta }}_{\mathrm{MLE}}\right)$ for the TILCI case and the TDLCI case, respectively. As expected, the dispersion in $\hat{\beta }$ was large when the number of specimens was relatively small. In contrast, the effect of starting LCI was relatively small and even appeared as though it acted as a negative factor for ${\mathrm{RLCI}}_{90\%}\left({\hat{\beta }}_{\mathrm{MLE}}\right)$ (as shown in Figure 13i). This counter-intuitive phenomenon could be caused by the low convergence ratio of estimation or by the reduction of possible combinations of random experimental results due to the wide starting LCI.

With respect to the case of ${\hat{\beta }}_{\mathrm{MRR}}$, the dispersion in ${\hat{\beta }}_{\mathrm{MRR}}$ was large when: (1) the number of specimens was small; (2) the value of ECF was low; and (3) TILCI scheme was applied (as shown in Figures S6 and S7). The results indicated that the effect of starting LCI on the ${\mathrm{RLCI}}_{90\%}\left({\hat{\beta }}_{\mathrm{MRR}}\right)$ was reversed between the range of 0.6 < ECF < 0.8. However, this was not a general tendency because strange valleys were also observed in both the ${\mathrm{RLCI}}_{90\%}\left({\hat{\beta }}_{\mathrm{MLE}}\right)$ and ${\mathrm{RLCI}}_{90\%}\left({\hat{\beta }}_{\mathrm{MRR}}\right)$ cases. It is necessary to examine this weird effect of LCI in future studies.

Additionally, critical lines were observed after which exceedingly wide ${\mathrm{RLCI}}_{90\%}\left(\hat{\beta }\right)$ were produced [12]. The gradients of ${\mathrm{RLCI}}_{90\%}\left(\hat{\beta }\right)$ were very high near the critical lines. The area of the critical region decreased when the TDLCI scheme was applied.

With respect to the MRR estimation, this critical region was a result of the inherent uncertainty of estimation because the convergence ratio for the MRR case was almost unity. Although the exact locations of the critical lines were not clear, it is recommended that experimenters should avoid this region.

The distributions of Weibull estimates, especially for $\hat{\beta }$, were not normal in most simulation cases. Therefore, the upper and lower bounds of the empirical confidence interval (e.g., ${\mathrm{RE}}_{5\%}$ and ${\mathrm{RE}}_{95\%}$, respectively) must be represented instead of ${\mathrm{RE}}_{50\%}$ and ${\mathrm{RLCI}}_{90\%}$.

**Figure 15:**
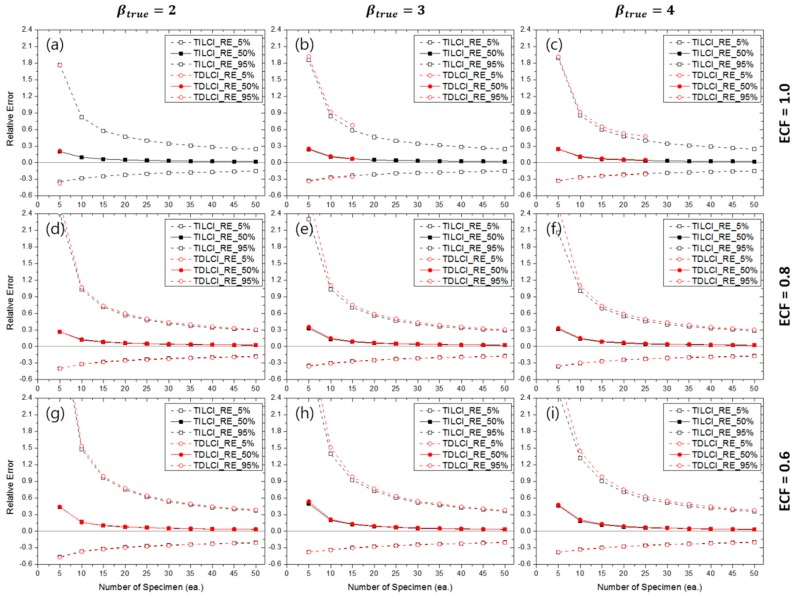
Effects of the number of specimens on $\mathrm{RE}\left({\hat{\beta }}_{\mathrm{MLE}}\right)$ for MNC = 10 lines when ECF = 1.0 and (**a**) $\beta_{\mathrm{true}}=2$, (**b**) $\beta_{\mathrm{true}}=3$, (**c**) $\beta_{\mathrm{true}}=4$; when ECF = 0.8 and (**d**) $\beta_{\mathrm{true}}=2$, (**e**) $\beta_{\mathrm{true}}=3$, (**f**) $\beta_{\mathrm{true}}=4$; when ECF = 0.6 and (**g**) $\beta_{\mathrm{true}}=2$, (**h**) $\beta_{\mathrm{true}}=3$, (**i**) $\beta_{\mathrm{true}}=4$.

Figure 15 shows the ${\mathrm{RE}}_{5\%}\left(\hat{\beta }\right)$, ${\mathrm{RE}}_{50\%}\left(\hat{\beta }\right)$ and ${\mathrm{RE}}_{95\%}\left(\hat{\beta }\right)$ for the TILCI and TDLCI cases when MNC = 10 and the MLE method were used for the estimation. Although the contour plots of ${\mathrm{RE}}_{50\%}\left({\hat{\beta }}_{\mathrm{MLE}}\right)$ and ${\mathrm{RLCI}}_{90\%}\left({\hat{\beta }}_{\mathrm{MLE}}\right)$ were quite complex, the estimation uncertainty (i.e., $\mathrm{RE}\left({\hat{\beta }}_{\mathrm{MLE}}\right)$) for the TILCI and TDLCI cases along the same MNC line were, interestingly, almost similar. This result is supported by the fact that the convergence ratios for the TILCI and TDLCI case were also similar along the same MNC line (as shown in Figure S3).

Similar to the MLE case, the $\mathrm{RE}\left({\hat{\beta }}_{\mathrm{MRR}}\right)$ for the TILCI and TDLCI cases along the same MNC line were almost the same (as shown in Figure S8). However, the overall uncertainty of MRR estimation slightly exceeded that of MLE estimation (when Figures 15 and Figure S8 were compared).

From this result, it is possible to calculate the empirical confidence interval and bias of the estimators when real cracking test conditions were given. For example, if the ECF of testing corresponded to 1.0 and the number of censoring during the test corresponded to 10 times, it could be estimated that the probability of obtaining $-0.22<\frac{{\hat{\beta }}_{\mathrm{MLE}}-\beta_{\mathrm{true}}}{\beta_{\mathrm{true}}}<0.50$ was approximately 90% and $\frac{{\hat{\beta }}_{\mathrm{MLE},\;50\%}-\beta_{\mathrm{true}}}{\beta_{\mathrm{true}}}≅0.053$ with 20 specimens when $\beta_{\mathrm{true}}=4$ was known (or estimated) for the testing material (as shown in Figure 15c).

### 4.4. Empirical Confidence Interval of $\hat{\eta }$

**Figure 16:**
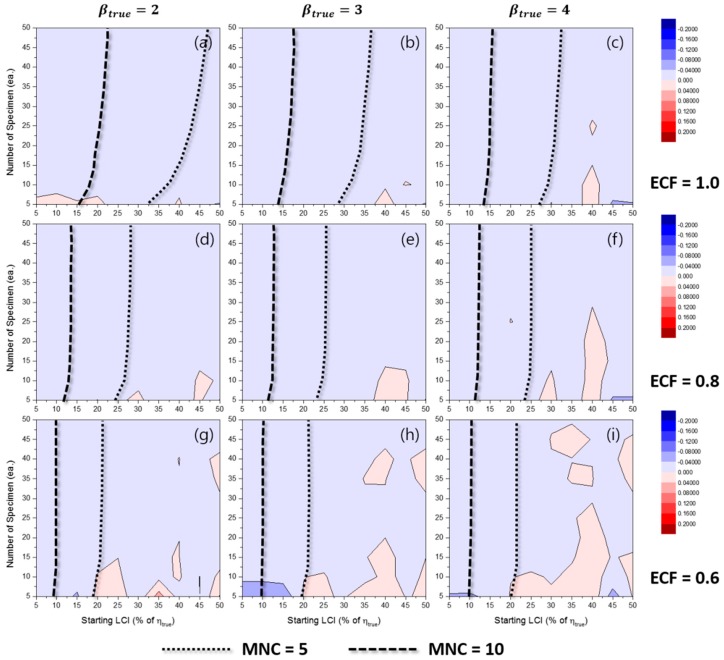
Effects of the number of specimens and starting LCI on ${\mathrm{RE}}_{50\%}\left({\hat{\eta }}_{\mathrm{MLE}}\right)$ for the TILCI case when ECF = 1.0 and (**a**) $\beta_{\mathrm{true}}=2$, (**b**) $\beta_{\mathrm{true}}=3$, (**c**) $\beta_{\mathrm{true}}=4$; when ECF = 0.8 and (**d**) $\beta_{\mathrm{true}}=2$, (**e**) $\beta_{\mathrm{true}}=3$, (**f**) $\beta_{\mathrm{true}}=4$; when ECF = 0.6 and (**g**) $\beta_{\mathrm{true}}=2$, (**h**) $\beta_{\mathrm{true}}=3$, (**i**) $\beta_{\mathrm{true}}=4$.

**Figure 17:**
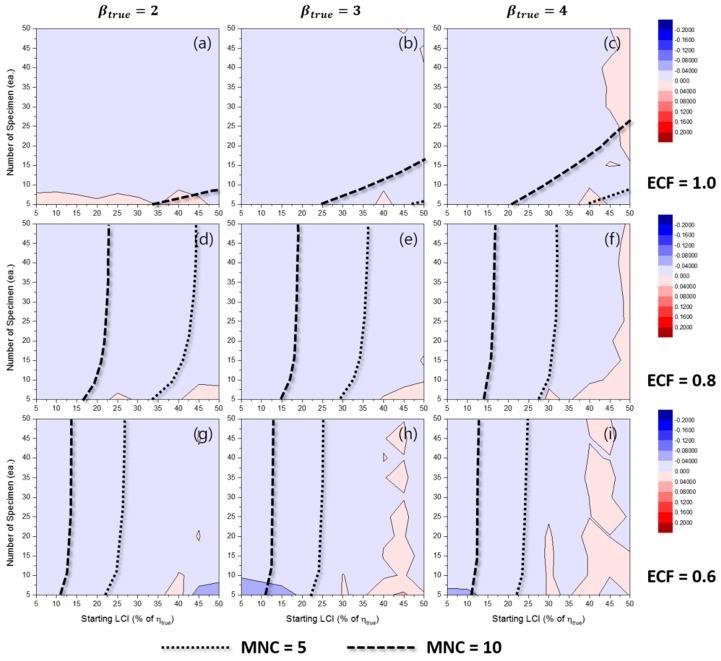
Effects of the number of specimens and starting LCI on ${\mathrm{RE}}_{50\%}\left({\hat{\eta }}_{\mathrm{MLE}}\right)$ for the TDLCI case when ECF = 1.0 and (**a**) $\beta_{\mathrm{true}}=2$, (**b**) $\beta_{\mathrm{true}}=3$, (**c**) $\beta_{\mathrm{true}}=4$; when ECF = 0.8 and (**d**) $\beta_{\mathrm{true}}=2$, (**e**) $\beta_{\mathrm{true}}=3$, (**f**) $\beta_{\mathrm{true}}=4$; when ECF = 0.6 and (**g**) $\beta_{\mathrm{true}}=2$, (**h**) $\beta_{\mathrm{true}}=3$, (**i**) $\beta_{\mathrm{true}}=4$.

Figure 16 and Figure 17 shows the contour plots of ${\mathrm{RE}}_{50\%}\left({\hat{\eta }}_{\mathrm{MLE}}\right)$ for the TILCI case and the TDLCI case, respectively. The results indicated that the value of ${\mathrm{RE}}_{50\%}\left({\hat{\eta }}_{\mathrm{MLE}}\right)$ was almost zero in every experimental condition. That is, with respect to the bias of estimation, ${\hat{\eta }}_{\mathrm{MLE}}$ was always reliable irrespective of the combination of experimental conditions in the simulation study.

However, it was likely that ${\hat{\eta }}_{\mathrm{MRR}}$ could be overestimated when: (1) the number of specimens was small; (2) the value of ECF was high; (3) the value of $\beta_{\mathrm{true}}$ was small; and (4) TILCI scheme was applied (see Figures S9 and S10). Nevertheless, the overall degree of bias was not significant when compared to the ${\hat{\eta }}_{\mathrm{MRR}}$ case.

**Figure 18:**
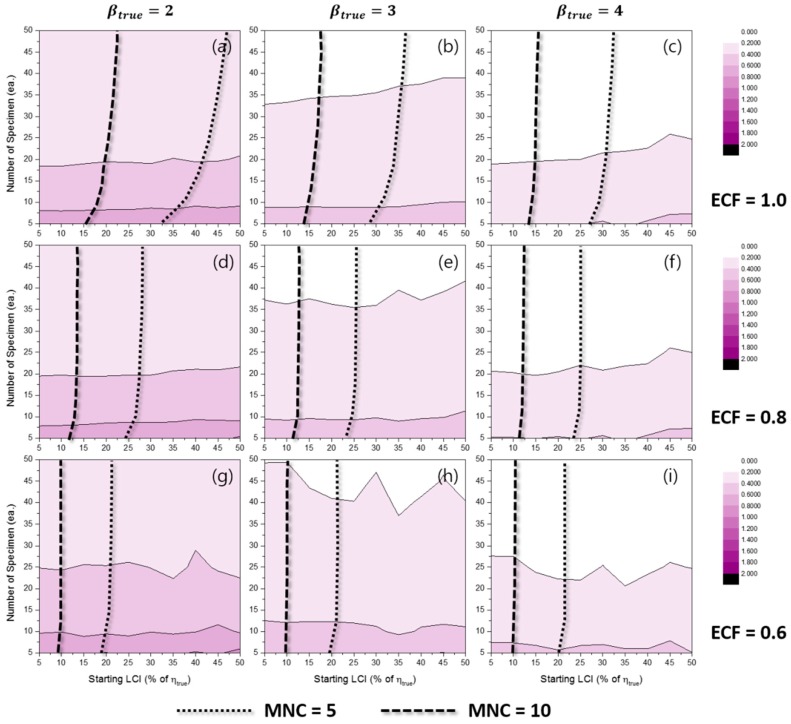
Effects of the number of specimens and starting LCI on ${\mathrm{RLCI}}_{90\%}\left({\hat{\eta }}_{\mathrm{MLE}}\right)$ for the TILCI case when ECF = 1.0 and (**a**) $\beta_{\mathrm{true}}=2$, (**b**) $\beta_{\mathrm{true}}=3$, (**c**) $\beta_{\mathrm{true}}=4$; when ECF = 0.8 and (**d**) $\beta_{\mathrm{true}}=2$, (**e**) $\beta_{\mathrm{true}}=3$, (**f**) $\beta_{\mathrm{true}}=4$; when ECF = 0.6 and (**g**) $\beta_{\mathrm{true}}=2$, (**h**) $\beta_{\mathrm{true}}=3$, (**i**) $\beta_{\mathrm{true}}=4$.

**Figure 19:**
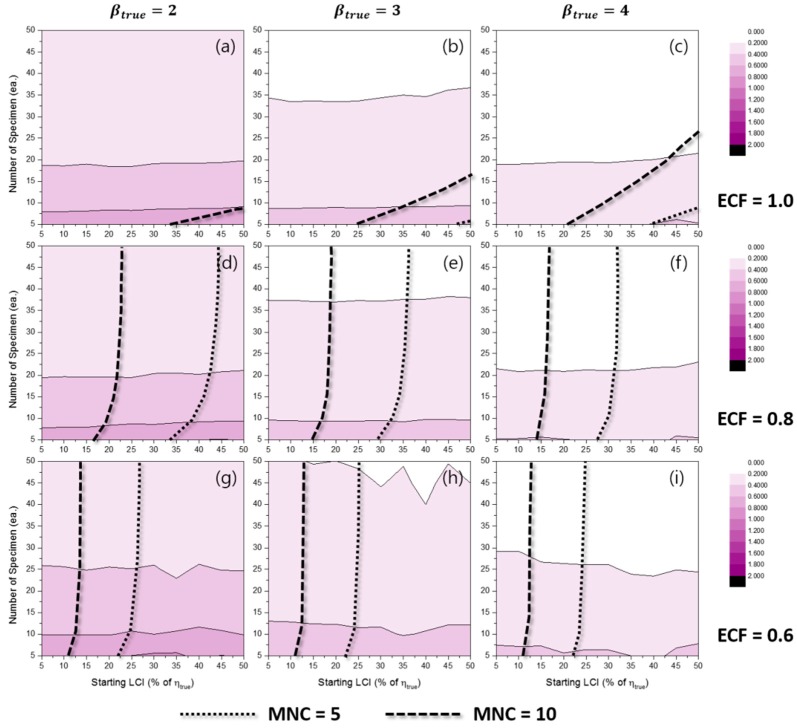
Effects of the number of specimens and starting LCI on ${\mathrm{RLCI}}_{90\%}\left({\hat{\eta }}_{\mathrm{MLE}}\right)$ for the TDLCI case when ECF = 1.0 and (**a**) $\beta_{\mathrm{true}}=2$, (**b**) $\beta_{\mathrm{true}}=3$, (**c**) $\beta_{\mathrm{true}}=4$; when ECF = 0.8 and (**d**) $\beta_{\mathrm{true}}=2$, (**e**) $\beta_{\mathrm{true}}=3$, (**f**) $\beta_{\mathrm{true}}=4$; when ECF = 0.6 and (**g**) $\beta_{\mathrm{true}}=2$, (**h**) $\beta_{\mathrm{true}}=3$, (**i**) $\beta_{\mathrm{true}}=4$.

Figure 18 and Figure 19 show the contour plots of ${\mathrm{RLCI}}_{90\%}\left({\hat{\eta }}_{\mathrm{MLE}}\right)$ for the TILCI case and the TDLCI case, respectively. When compared to the case of ${\mathrm{RLCI}}_{90\%}\left({\hat{\beta }}_{\mathrm{MLE}}\right)$, the overall value of ${\mathrm{RLCI}}_{90\%}\left({\hat{\eta }}_{\mathrm{MLE}}\right)$ was quite small. In other words, the results indicated more precise ${\hat{\eta }}_{\mathrm{MLE}}$ relative to ${\hat{\beta }}_{\mathrm{MLE}}$ under the same experimental conditions [12]. The dispersion in ${\hat{\eta }}_{\mathrm{MLE}}$ was large when: (1) the number of specimens was small; (2) the value of ECF was low; and (3) the value of $\beta_{\mathrm{true}}$ was small. In the simulation results, the starting LCI and TDLCI application did not affect ${\mathrm{RLCI}}_{90\%}\left({\hat{\beta }}_{\mathrm{MLE}}\right)$.

With respect to the case of ${\hat{\eta }}_{\mathrm{MRR}}$, in a manner similar to the MLE case, the results indicated more precise ${\hat{\eta }}_{\mathrm{MRR}}$ relative to ${\hat{\beta }}_{\mathrm{MRR}}$ (as shown in Figures S11 and S12). The dispersion in ${\hat{\eta }}_{\mathrm{MRR}}$ was large when: (1) the number of specimens was small; (2) the value of ECF was low; and (3) the value of $\beta_{\mathrm{true}}$ was small. It is shown that in a manner similar to the ${\hat{\beta }}_{\mathrm{MRR}}$ case, the effect of the starting LCI was reversed between the range of 0.6 < ECF < 0.8. This could be due to the reduction of possible combinations of simulation results, as previously mentioned. Additionally, this was not a general tendency given the occurrence of the strange valleys (as shown in Figure S11h).

The critical regions did not appear in the cases of both ${\mathrm{RLCI}}_{90\%}\left({\hat{\eta }}_{\mathrm{MLE}}\right)$ and ${\mathrm{RLCI}}_{90\%}\left({\hat{\eta }}_{\mathrm{MRR}}\right)$.

**Figure 20:**
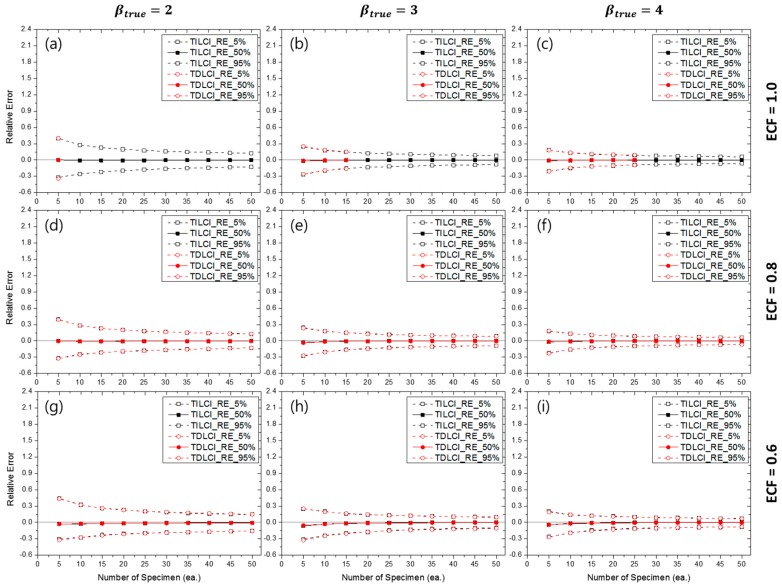
Effects of the number of specimens on $\mathrm{RE}\left({\hat{\eta }}_{\mathrm{MLE}}\right)$ for MNC = 10 lines when ECF = 1.0 and (**a**) $\beta_{\mathrm{true}}=2$, (**b**) $\beta_{\mathrm{true}}=3$, (**c**) $\beta_{\mathrm{true}}=4$; when ECF = 0.8 and (**d**) $\beta_{\mathrm{true}}=2$, (**e**) $\beta_{\mathrm{true}}=3$, (**f**) $\beta_{\mathrm{true}}=4$; when ECF = 0.6 and (**g**) $\beta_{\mathrm{true}}=2$, (**h**) $\beta_{\mathrm{true}}=3$, (**i**) $\beta_{\mathrm{true}}=4$.

Figure 20 shows the comparison of the TILCI and TDLCI cases for $\mathrm{RE}\left({\hat{\eta }}_{\mathrm{MLE}}\right)$ when MNC = 10. Like the ${\hat{\beta }}_{\mathrm{MLE}}$ cases, the $\mathrm{RE}\left({\hat{\eta }}_{\mathrm{MLE}}\right)$ for the TILCI and TDLCI cases along the same MNC line was almost the same.

Similar to the other cases, the values of $\mathrm{RE}\left({\hat{\eta }}_{\mathrm{MRR}}\right)$ for the TILCI and TDLCI cases along the same MNC line were also similar (see Figure S13). This phenomenon implied that the effect of time dependence on LCI was not very critical when the equivalent MNC condition was applied. The overall uncertainty of ${\hat{\eta }}_{\mathrm{MRR}}$ was slightly larger than that of ${\hat{\eta }}_{\mathrm{MLE}}$ (when Figure 20 and Figure S13 were compared).

## 5. Conclusions

The main goal of this study included deriving quantitative estimation uncertainties for experimenters developing a Weibull distribution model via cracking tests. The widely-used MRR and MLE methods were performed with respect to the Weibull estimation. Monte Carlo simulations were used to quantify uncertainties of MRR and ML estimators in various experimental conditions by considering the effects of: (1) true Weibull parameters; (2) the number of specimens; (3) end cracking fractions; (4) starting LCI; and (5) the time dependence of LCI. The following conclusions were drawn from the study:

- The Weibull distribution was appropriate for the statistical model of cracking time at a macroscopic scale.
- The application of the same MNC line was reasonable as a criterion of uncertainty comparison between the TILCI and TDLCI cases. In this criterion, there was interestingly no (or very little) difference between the estimation uncertainty with the TILCI scheme and that with the TDLCI scheme.
- The branch point of the ECF with respect to the value of $\beta_{\mathrm{true}}$ on MTD is suspected to be 0.632 (=F(η)). For the case when the value of ECF was relatively low, a non-consistent effect of starting LCI on MTD was observed.
- In most cases, ${\hat{\beta }}_{\mathrm{MLE}}$ and ${\hat{\beta }}_{\mathrm{MRR}}$ showed a tendency to be overestimated and dispersed when the number of specimens was small and the value of ECF was low. It was difficult to find the general effect of $\beta_{\mathrm{true}}$ and starting LCI due to the occurrence of strange valleys. It was shown that there were critical regions in which the estimators, whose dispersions were extremely large, were produced. Thus, the study recommends that experimenters should avoid this region.
- ${\hat{\eta }}_{\mathrm{MLE}}$ showed almost zero bias in all simulation ranges. Conversely, ${\hat{\eta }}_{\mathrm{MRR}}$ could be overestimated in some cases. Both ${\hat{\eta }}_{\mathrm{MLE}}$ and ${\hat{\eta }}_{\mathrm{MRR}}$ tended to be dispersed when: (1) the number of specimens was small; (2) the value of ECF was small; and (3) the value of $\beta_{\mathrm{true}}$ was small. In most cases, the starting LCI did not affect the estimation uncertainty of $\hat{\eta }$.
- The overall bias and dispersion of $\hat{\eta }$ were much lower than those of $\hat{\beta }$ in the simulation study range.

## Figures and Tables

**Table 1:** Example of the random simulation data with the time-dependent length of censoring interval (TDLCI) scheme (number of specimens: 10; starting LCI: 40% of $\eta_{\mathrm{true}}$; end cracking fraction (ECF): 1.0; $\beta_{\mathrm{true}}$: 3.0).

**Number of Survived Specimen**	10		10		6		3		3		2		0
**Survived Fraction**	1.0		1.0		0.6		0.3		0.3		0.2		0
**LCI (% of** $\eta_{\mathrm{true}}$**)**		40		40 (=40 × 1.0)		24 (=40 × 0.6)		12 (=40 × 0.3)		12 (=40 × 0.3)		8 (=40 × 0.2)	
**Censoring Time (% of** $\eta_{\mathrm{true}}$**)**	0		40 (=0 + 40)		80 (=40 + 40)		104 (=80 + 24)		116 (=104 + 12)		128 (=116 + 12)		136 (=128 + 8)

**Table 2:** Experimental factors considered in the Monte Carlo simulation.

True Weibull Parameters		Number of Specimens	ECF	LCI	
$\eta_{\mathrm{true}}$ (Dimensionless Time)	$\beta_{\mathrm{true}}$			Starting LCI (% of $\eta_{\mathrm{true}}$)	Time Dependence of LCI
100	2	5	0.6	5	Time independent
-	3	10	0.8	10	Time dependent
-	4	15	1.0	15	-
-	-	20	-	20	-
-	-	25	-	25	-
-	-	30	-	30	-
-	-	35	-	35	-
-	-	40	-	40	-
-	-	45	-	45	-
-	-	50	-	50	-

**Table 3:** An example of the combination of experimental factors.

True Weibull Parameters		Number of Specimens	ECF	LCI	
$\eta_{\mathrm{true}}$ (Dimensionless Time)	$\beta_{\mathrm{true}}$			Starting LCI (% of $\eta_{\mathrm{true}}$)	Time Dependence of LCI
100	3	10	1.0	40	Time dependent

## Supplementary Materials

The following are available online at www.mdpi.com/1996-1944/10/1/3/s1: (1) convergence ratio of numerical estimation; (2) empirical confidence interval of ${\hat{\beta }}_{\mathrm{MRR}}$; and (3) empirical confidence interval of ${\hat{\eta }}_{\mathrm{MRR}}$.
