# Supplementary material for: Effects of Cracking Test Conditions on Estimation Uncertainty for Weibull Parameters Considering Time-Dependent Censoring Interval
Source: Materials (Basel). 2016 Dec 23;10(1):3. doi: 10.3390/ma10010003 (PMC5344630; doi:10.3390/ma10010003)
Supplement: Supplementary file 1 [file materials-10-00003-s001.pdf]

Supplementary Materials: Effects of Cracking Test Conditions on Estimation Uncertainty for Weibull Parameters Considering Time-Dependent Censoring Interval

Jae Phil Park, Chanseok Park, Jongwon Cho and Chi Bum Bahn

1. Convergence Ratio

Figures S1 and S2 represent the effects of experimental conditions on the convergence ratio of MLE, which was numerically performed by the fsolve function in MATLAB. The convergence ratio for a certain experimental case is defined as follows:

$$\text{Convergence ratio} = \frac{Number\ of\ converged\ estimations\ by\ numerical\ solver}{Number\ of\ replicates(= 20{,}000)}. \qquad \text{(S1)}$$

If the convergence ratio was too low, there would be a filtering effect, which was caused by the disregard of outlier estimators [1]. That is, for a low convergence ratio region, output estimators were not purely random. It is recommended to be careful when analyzing the results in this region. For the case of MRR estimation, the convergence ratio reached almost unity for all experimental conditions.

Although it is noted that the expectation-maximization (EM) sequences can overcome this non-convergence issue for MLE, which occurred when the other numerical methods (e.g., the Newton–Raphson method) are used to estimate, a complete EM sequence for interval-censored Weibull data has not been suggested until now, even for the recent study covering this issue [2].

From the simulation results (see Figures S1 and S2), it is shown that the convergence ratio decreased with: (1) a small number of specimens; (2) a wide starting LCI; (3) a low value of ECF; (4) a high value of β_{true}; and (5) the application of the TILCI scheme. However, for the case when the values of ECF and β_{true} are relatively low, there is a weird tendency of a decreasing convergence ratio with narrowing starting LCI (see Figures S1g and S2g). Further study is needed to interpret this phenomenon.

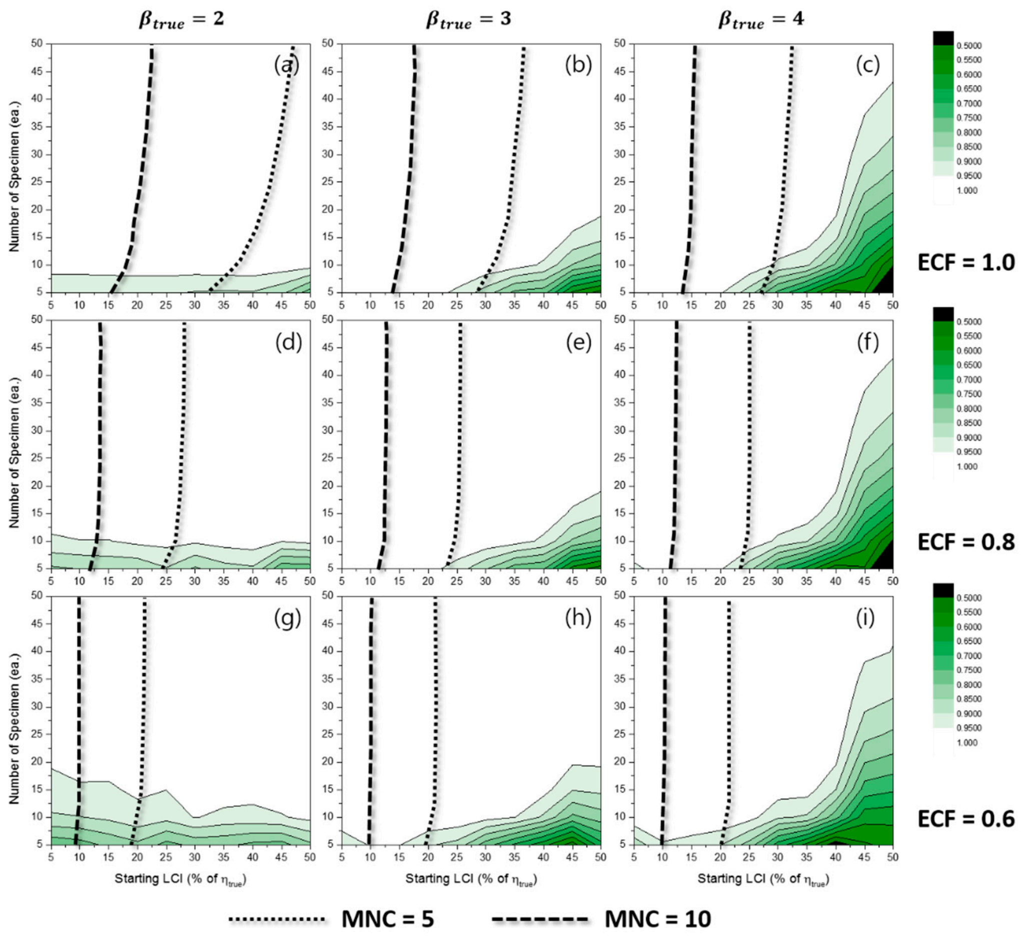

Figure S1. Effects of the number of specimens and starting LCI on the convergence ratio for the TILCI case when ECF = 1.0 and (**a**) $\beta_{true} = 2$, (**b**) $\beta_{true} = 3$, (**c**) $\beta_{true} = 4$; when ECF = 0.8 and (**d**) $\beta_{true} = 2$, (**e**) $\beta_{true} = 3$, (**f**) $\beta_{true} = 4$; when ECF = 0.6 and (**g**) $\beta_{true} = 2$, (**h**) $\beta_{true} = 3$, (**i**) $\beta_{true} = 4$.

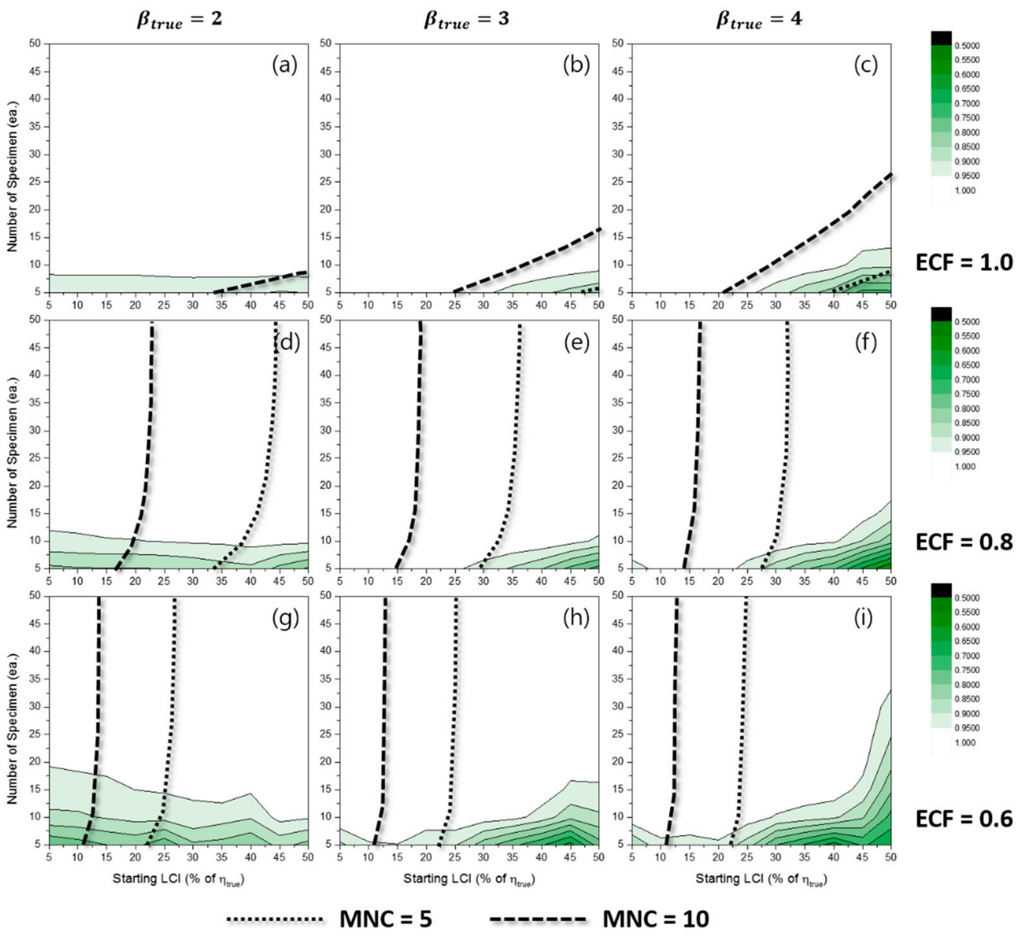

Figure S2. Effects of the number of specimens and starting LCI on the convergence ratio for the TDLCI case when ECF = 1.0 and (**a**) $\beta_{true} = 2$, (**b**) $\beta_{true} = 3$, (**c**) $\beta_{true} = 4$; when ECF = 0.8 and (**d**) $\beta_{true} = 2$, (**e**) $\beta_{true} = 3$, (**f**) $\beta_{true} = 4$; when ECF = 0.6 and (**g**) $\beta_{true} = 2$, (**h**) $\beta_{true} = 3$, (**i**) $\beta_{true} = 4$.

In the comparison between the TILCI and TDLCI cases, the TILCI case returns a low convergence ratio when the other experimental factors are the same, especially for the wide starting LCI. However, the TDLCI case requires much more MNC. To compensate this effect, Figure S3 compares the effect of the experimental conditions on the convergence ratio when the same MNC line (i.e., MNC = 10 in this case) is applied as a criterion. Then, the result shows, interestingly, almost the same convergence ratio between the TILCI and TDLCI cases.

Though the convergence ratio in Figure S3g is relatively low, we assume that there is no filtering effect when the MNC = 10 is applied as a criterion.

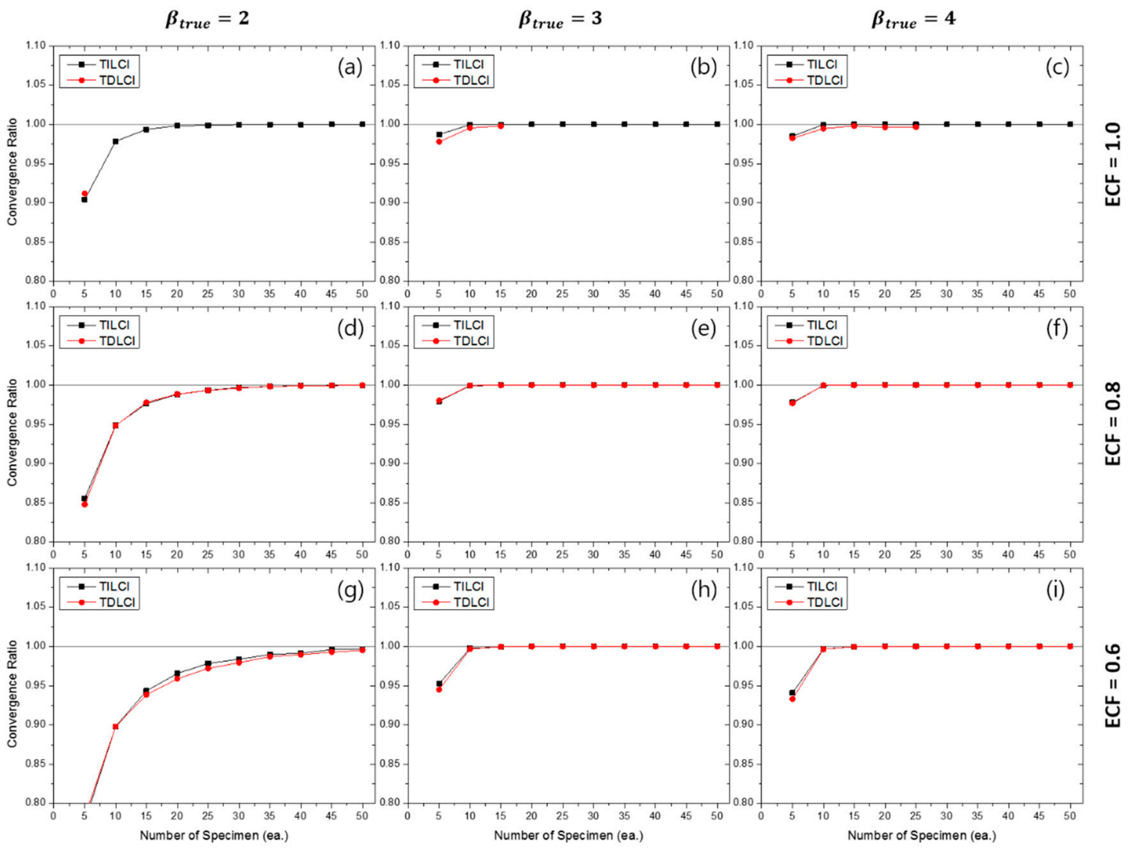

Figure S3. Effects of the number of specimens on the convergence ratio for MNC = 10 lines when ECF = 1.0 and (**a**) $\beta_{true} = 2$, (**b**) $\beta_{true} = 3$, (**c**) $\beta_{true} = 4$; when ECF = 0.8 and (**d**) $\beta_{true} = 2$, (**e**) $\beta_{true} = 3$, (**f**) $\beta_{true} = 4$; when ECF = 0.6 and (**g**) $\beta_{true} = 2$, (**h**) $\beta_{true} = 3$, (**i**) $\beta_{true} = 4$.

2. Empirical Confidence Interval of $\hat{\beta}_{MRR}$

The important difference between the MLE and MRR is that the convergence ratio for MRR estimation is almost unity in every experimental combination. Thus, we can insist that there would be no filtering effect for the MRR cases.

Figure S4 shows the contour plots of the $RE_{50\%}(\hat{\beta}_{MRR})$ for the TILCI case, and Figure S5 shows those for the TDLCI case. You can see the detailed discussion of the figures below in the main text of the paper.

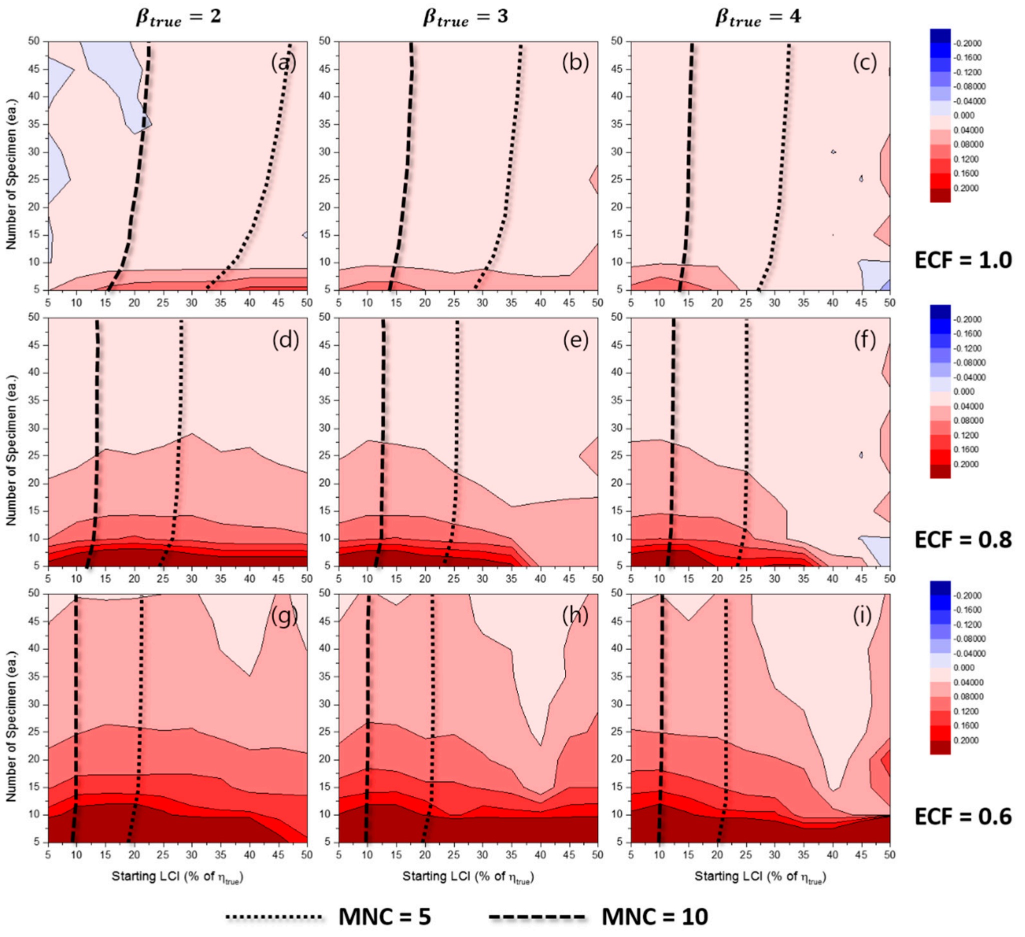

Figure S4. Effects of the number of specimens and starting LCI on $RE_{50\%}(\hat{\beta}_{MRR})$ for the TILCI case when ECF = 1.0 and (**a**) β_{true} = 2, (**b**) β_{true} = 3, (**c**) β_{true} = 4; when ECF = 0.8 and (**d**) β_{true} = 2, (**e**) β_{true} = 3, (**f**) β_{true} = 4; when ECF = 0.6 and (**g**) β_{true} = 2, (**h**) β_{true} = 3, (**i**) β_{true} = 4.

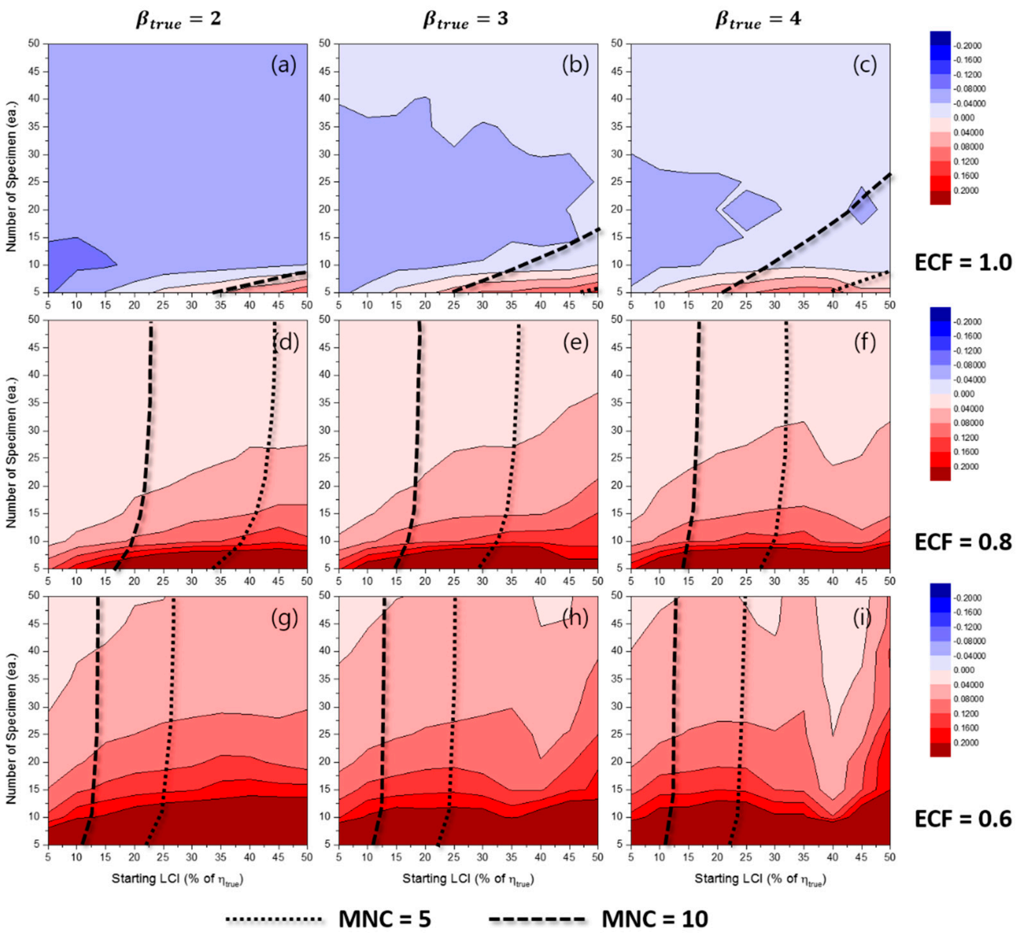

Figure S5. Effects of the number of specimens and starting LCI on $RE_{50\%}(\hat{\beta}_{MRR})$ for the TDLCI case when ECF = 1.0 and (**a**) β_{true} = 2, (**b**) β_{true} = 3, (**c**) β_{true} = 4; when ECF = 0.8 and (**d**) β_{true} = 2, (**e**) β_{true} = 3, (**f**) β_{true} = 4; when ECF = 0.6 and (**g**) β_{true} = 2, (**h**) β_{true} = 3, (**i**) β_{true} = 4.

Figure S6 shows the contour plots of $RLCI_{90\%}(\hat{\beta}_{MRR})$ for the TILCI case, and Figure S7 shows those for the TDLCI case.

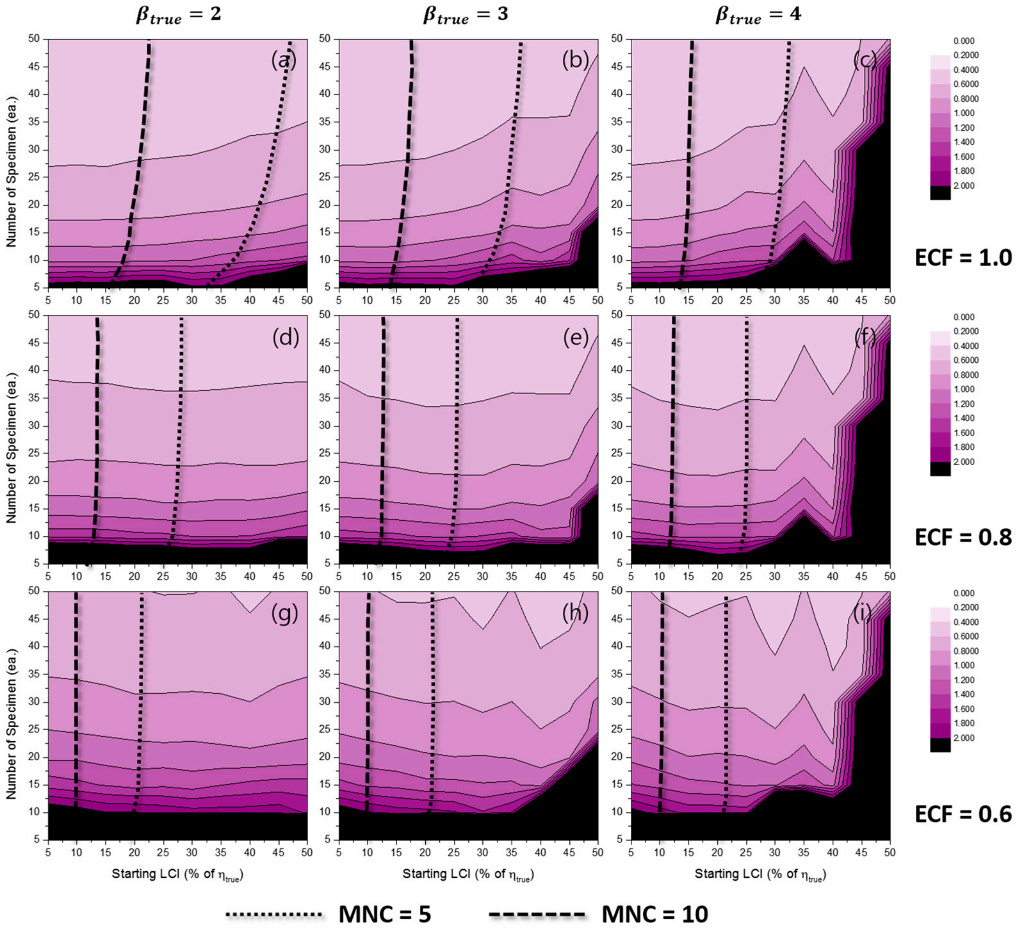

Figure S6. Effects of the number of specimens and starting LCI on $RLCI_{90\%}(\hat{\beta}_{MRR})$ for the TILCI case when ECF = 1.0 and (**a**) β_{true} = 2, (**b**) β_{true} = 3, (**c**) β_{true} = 4; when ECF = 0.8 and (**d**) β_{true} = 2, (**e**) β_{true} = 3, (**f**) β_{true} = 4; when ECF = 0.6 and (**g**) β_{true} = 2, (**h**) β_{true} = 3, (**i**) β_{true} = 4.

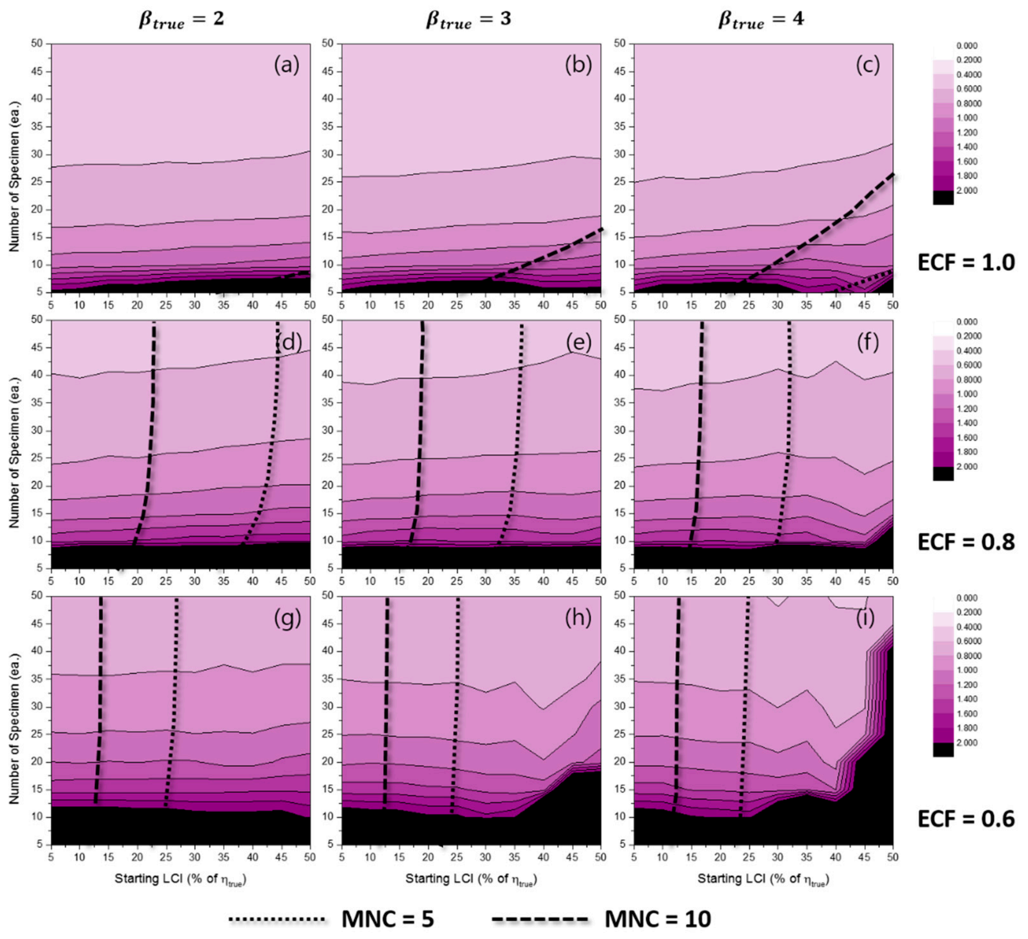

Figure S7. Effects of the number of specimens and starting LCI on $RLCI_{90\%}(\hat{\beta}_{MRR})$ for the TDLCI case when ECF = 1.0 and (**a**) $\beta_{true} = 2$, (**b**) $\beta_{true} = 3$, (**c**) $\beta_{true} = 4$; when ECF = 0.8 and (**d**) $\beta_{true} = 2$, (**e**) $\beta_{true} = 3$, (**f**) $\beta_{true} = 4$; when ECF = 0.6 and (**g**) $\beta_{true} = 2$, (**h**) $\beta_{true} = 3$, (**i**) $\beta_{true} = 4$.

Figure S8 shows the comparison of the TILCI and TDLCI cases for RE($\hat{\beta}_{MRR}$) when MNC = 10.

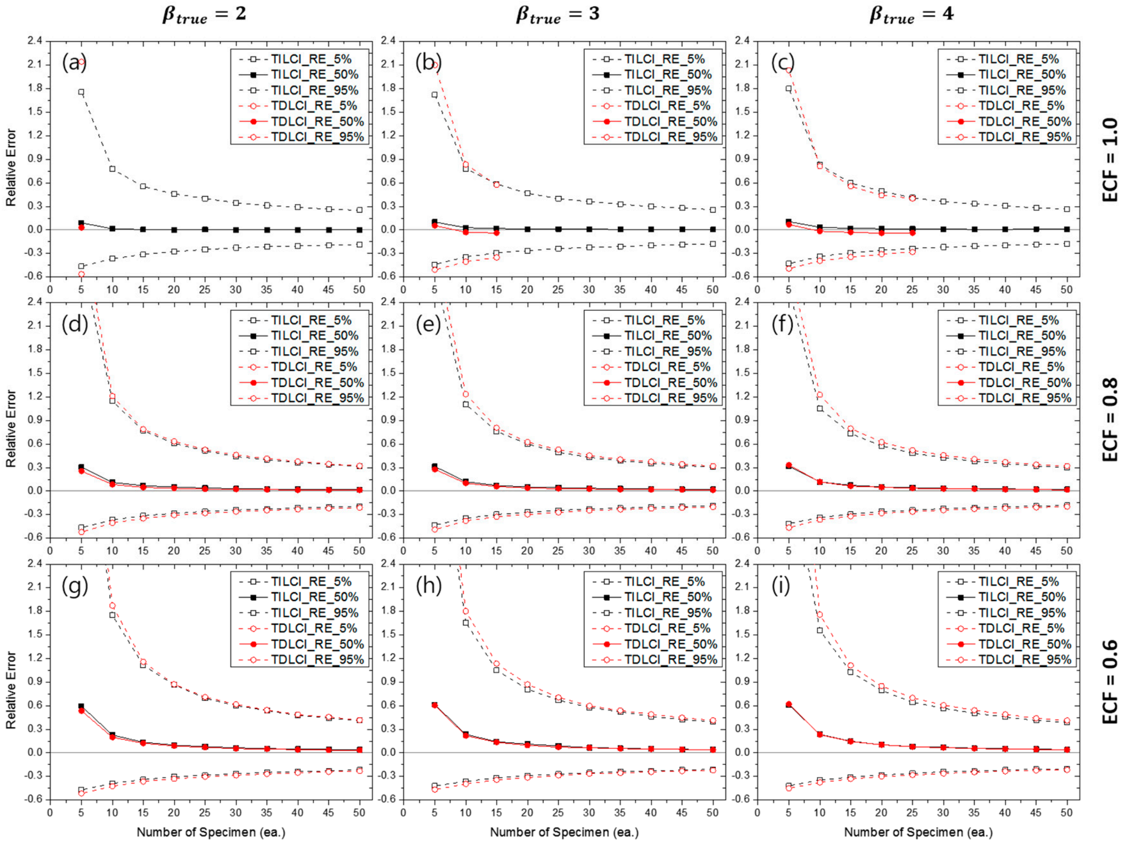

Figure S8. Effects of the number of specimens on RE($\hat{\beta}_{MRR}$) for MNC = 10 lines when ECF = 1.0 and (**a**) $\beta_{true} = 2$, (**b**) $\beta_{true} = 3$, (**c**) $\beta_{true} = 4$; when ECF = 0.8 and (**d**) $\beta_{true} = 2$, (**e**) $\beta_{true} = 3$, (**f**) $\beta_{true} = 4$; when ECF = 0.6 and (**g**) $\beta_{true} = 2$, (**h**) $\beta_{true} = 3$, (**i**) $\beta_{true} = 4$.

3. Empirical Confidence Interval of $\hat{\eta}_{MRR}$

Figure S9 shows the contour plots of $RE_{50\%}(\hat{\eta}_{MRR})$ for the TILCI case, and Figure S10 shows those for the TDLCI case. You can see the detailed discussion of the figures below in the main text of the paper.

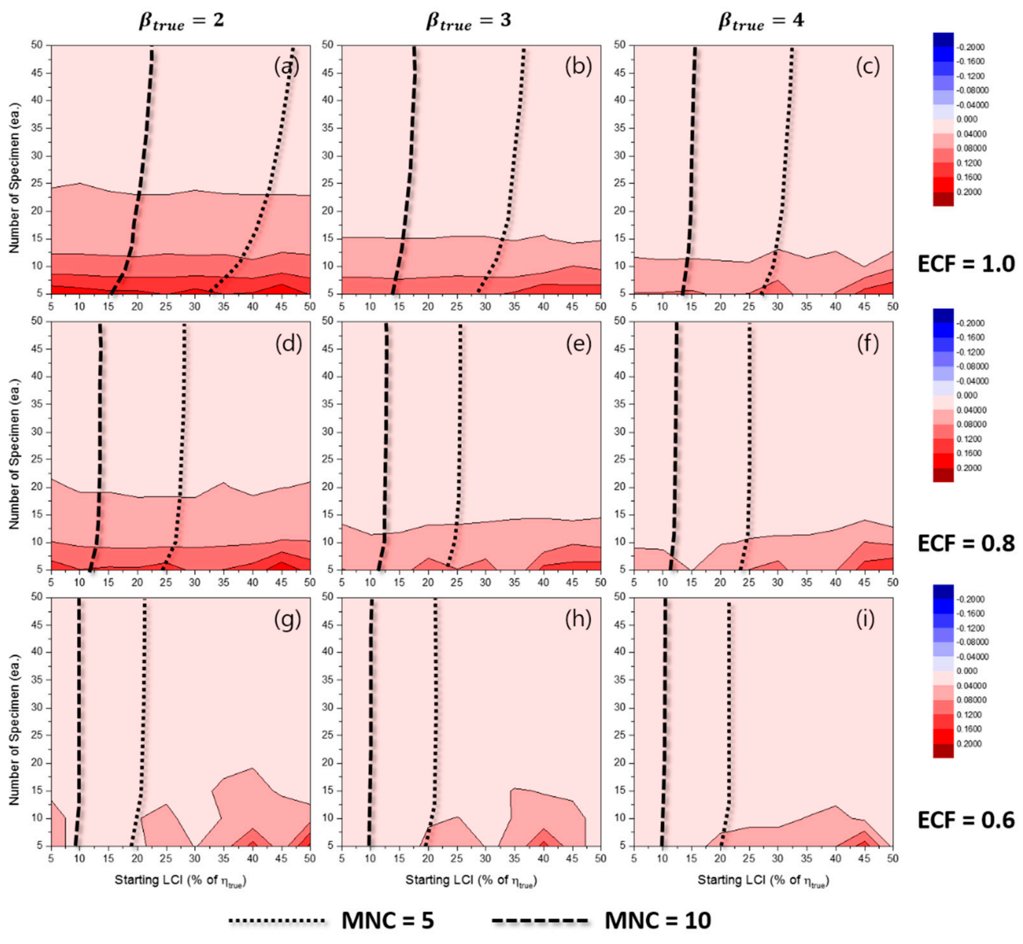

Figure S9. Effects of the number of specimens and starting LCI on $RE_{50\%}(\hat{\eta}_{MRR})$ for the TILCI case when ECF = 1.0 and (**a**) $\beta_{true} = 2$, (**b**) $\beta_{true} = 3$, (**c**) $\beta_{true} = 4$; when ECF = 0.8 and (**d**) $\beta_{true} = 2$, (**e**) $\beta_{true} = 3$, (**f**) $\beta_{true} = 4$; when ECF = 0.6 and (**g**) $\beta_{true} = 2$, (**h**) $\beta_{true} = 3$, (**i**) $\beta_{true} = 4$.

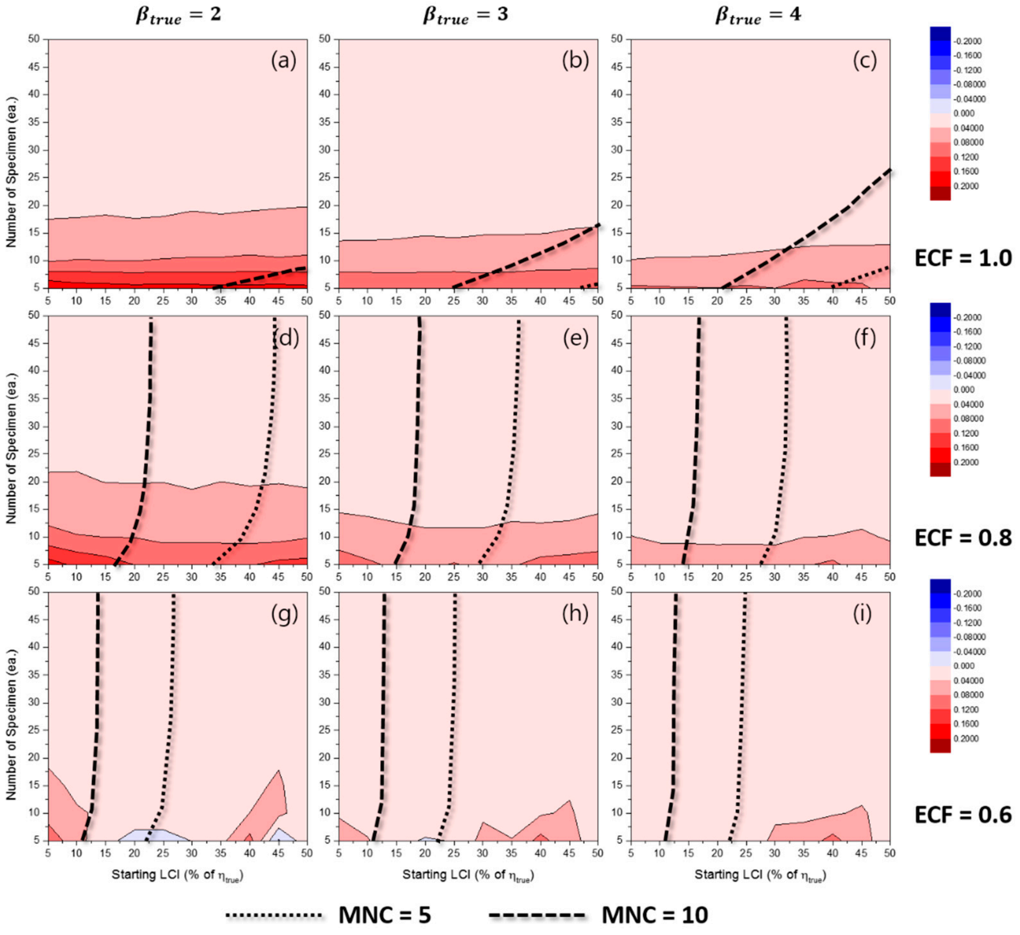

Figure S10. Effects of the number of specimens and starting LCI on $RE_{50\%}(\hat{\eta}_{MRR})$ for the TDLCI case when ECF = 1.0 and (**a**) β_{true} = 2, (**b**) β_{true} = 3, (**c**) β_{true} = 4; when ECF = 0.8 and (**d**) β_{true} = 2, (**e**) β_{true} = 3, (**f**) β_{true} = 4; when ECF = 0.6 and (**g**) β_{true} = 2, (**h**) β_{true} = 3, (**i**) β_{true} = 4.

Figure S11 shows the contour plots of $RLCI_{90\%}(\hat{\eta}_{MRR})$ for the TILCI case, and Figure S12 shows those for the TDLCI case.

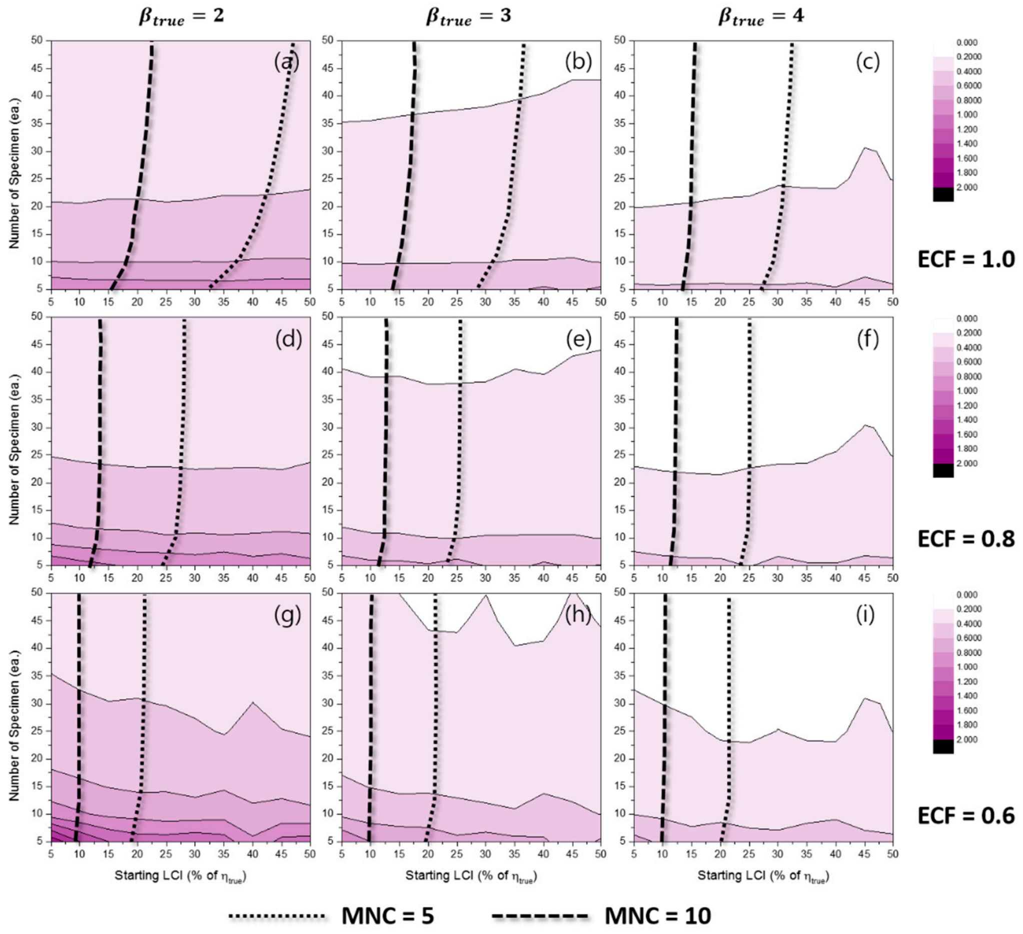

Figure S11. Effects of the number of specimens and starting LCI on $RLCI_{90\%}(\hat{\eta}_{MRR})$ for the TILCI case when ECF = 1.0 and (**a**) $\beta_{true} = 2$, (**b**) $\beta_{true} = 3$, (**c**) $\beta_{true} = 4$; when ECF = 0.8 and (**d**) $\beta_{true} = 2$, (**e**) $\beta_{true} = 3$, (**f**) $\beta_{true} = 4$; when ECF = 0.6 and (**g**) $\beta_{true} = 2$, (**h**) $\beta_{true} = 3$, (**i**) $\beta_{true} = 4$.

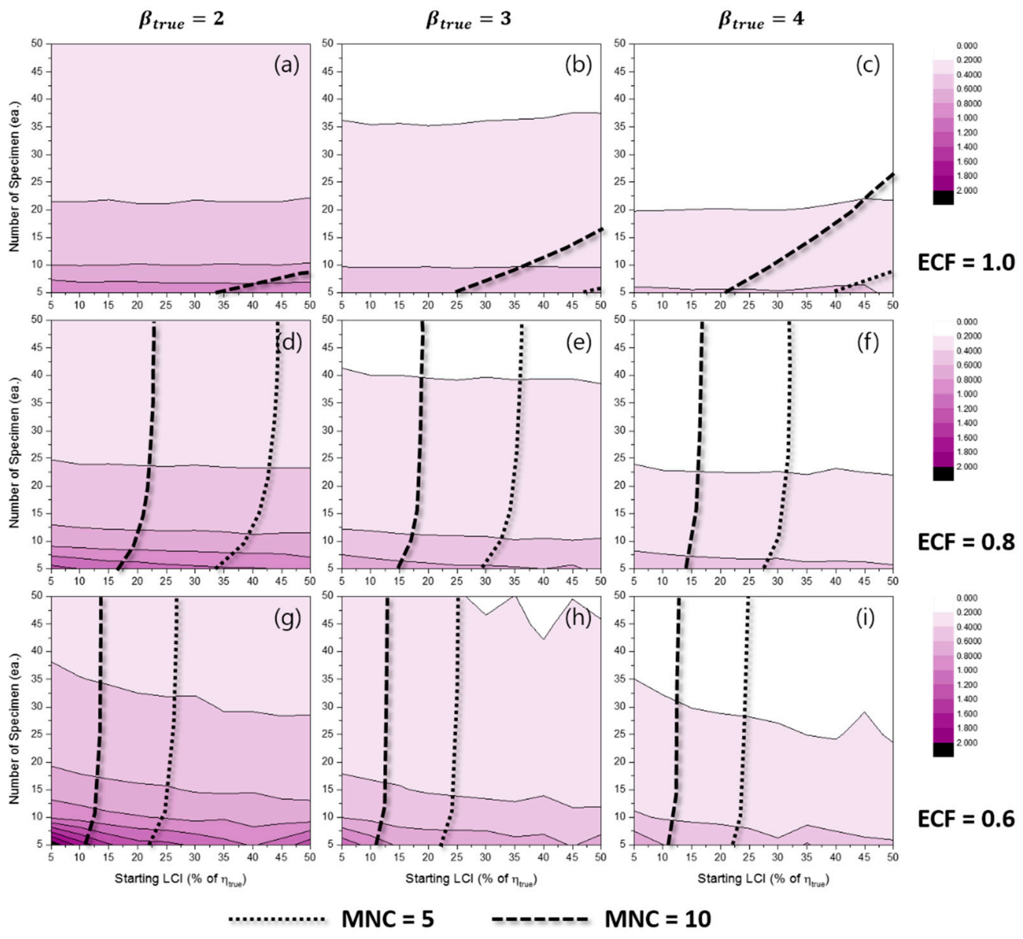

Figure S12. Effects of the number of specimens and starting LCI on $RLCI_{90\%}(\hat{\eta}_{MRR})$ for the TDLCI case when ECF = 1.0 and (**a**) $\beta_{true} = 2$, (**b**) $\beta_{true} = 3$, (**c**) $\beta_{true} = 4$; when ECF = 0.8 and (**d**) $\beta_{true} = 2$, (**e**) $\beta_{true} = 3$, (**f**) $\beta_{true} = 4$; when ECF = 0.6 and (**g**) $\beta_{true} = 2$, (**h**) $\beta_{true} = 3$, (**i**) $\beta_{true} = 4$.

Figure S13 shows the comparison of the TILCI and TDLCI cases for RE($\hat{\eta}_{MRR}$) when MNC = 10.

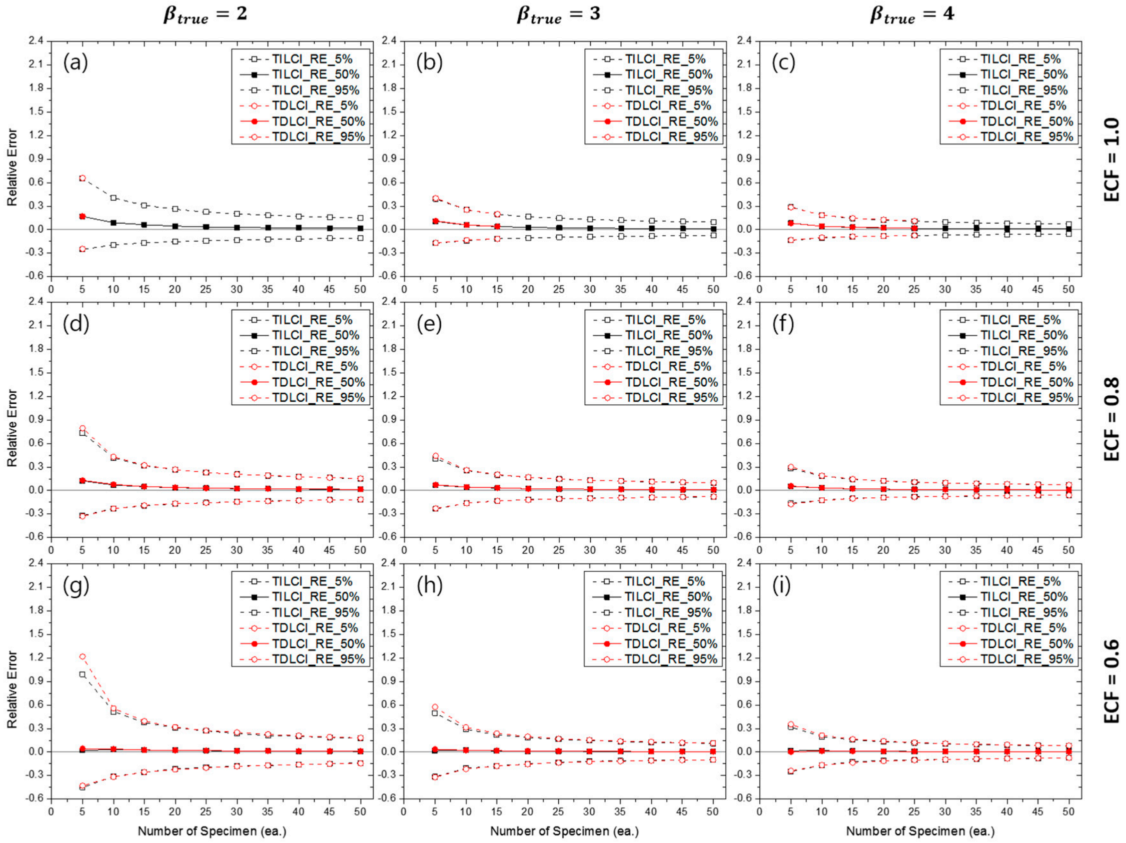

Figure S13. Effects of the number of specimens on RE($\hat{\eta}_{MRR}$) for MNC = 10 lines when ECF = 1.0 and (**a**) $\beta_{true} = 2$, (**b**) $\beta_{true} = 3$, (**c**) $\beta_{true} = 4$; when ECF = 0.8 and (**d**) $\beta_{true} = 2$, (**e**) $\beta_{true} = 3$, (**f**) $\beta_{true} = 4$; when ECF = 0.6 and (**g**) $\beta_{true} = 2$, (**h**) $\beta_{true} = 3$, (**i**) $\beta_{true} = 4$.

References

1. Park, J.P.; Bahn, C.B. Uncertainty Evaluation of Weibull Estimators through Monte Carlo Simulation: Applications for Crack Initiation Testing. *Materials* **2016**, *9*, 521.
2. Pradhan, B.; Kundu, D. Analysis of Interval-Censored Data with Weibull Lifetime Distribution. *Sankhya B* **2014**, *76*, 120–139.
